# Parallel derivation of isogenic human primed and naive induced pluripotent stem cells

**DOI:** 10.1038/s41467-017-02107-w

**Published:** 2018-01-24

**Authors:** Stéphanie Kilens, Dimitri Meistermann, Diego Moreno, Caroline Chariau, Anne Gaignerie, Arnaud Reignier, Yohann Lelièvre, Miguel Casanova, Céline Vallot, Steven Nedellec, Léa Flippe, Julie Firmin, Juan Song, Eric Charpentier, Jenna Lammers, Audrey Donnart, Nadège Marec, Wallid Deb, Audrey Bihouée, Cédric Le Caignec, Claire Pecqueur, Richard Redon, Paul Barrière, Jérémie Bourdon, Vincent Pasque, Magali Soumillon, Tarjei S. Mikkelsen, Claire Rougeulle, Thomas Fréour, Laurent David, The Milieu Intérieur Consortium, Laurent Abel, Andres Alcover, Kalla Astrom, Philippe Bousso, Pierre Bruhns, Ana Cumano, Darragh Duffy, Caroline Demangel, Ludovic Deriano, James Di Santo, Françoise Dromer, Gérard Eberl, Jost Enninga, Jacques Fellay, Antonio Freitas, Odile Gelpi, Ivo Gomperts-Boneca, Serge Hercberg, Olivier Lantz, Claude Leclerc, Hugo Mouquet, Etienne Patin, Sandra Pellegrini, Stanislas Pol, Lars Rogge, Anavaj Sakuntabhai, Olivier Schwartz, Benno Schwikowski, Spencer Shorte, Vassili Soumelis, Frédéric Tangy, Eric Tartour, Antoine Toubert, Marie-Noëlle Ungeheuer, Lluis Quintana-Murci, Matthew L. Albert

**Affiliations:** 1 Centre de Recherche en Transplantation et Immunologie UMR1064, INSERM, Université de Nantes, Nantes, France; 2 Institut de Transplantation Urologie Néphrologie (ITUN), CHU Nantes, Nantes, France; 3 LabEx IGO “Immunotherapy, Graft, Oncology”, Nantes, France; 4 Laboratoire des Sciences du Numérique de Nantes, LS2N, UMR CNRS 6004, Université de Nantes, Nantes, France; 5 INSERM UMS 016, SFR Francois Bonamy, iPSC Core Facility, Nantes, France; CNRS, UMS 3556, Nantes, France; Université de Nantes, Nantes, France; CHU Nantes, Nantes, France; 6 CHU Nantes, Service de Biologie de la Reproduction, Nantes, France; 7 Sorbonne Paris Cité, Epigenetics and Cell Fate, UMR 7216 CNRS, Université Paris Diderot, Paris, France; 8 INSERM UMS 016, SFR Francois Bonamy, MicroPicell Core Facility, Nantes, France; CNRS, UMS 3556, Nantes, France; Université de Nantes, Nantes, France; CHU de Nantes, Nantes, France; 9 KU Leuven–University of Leuven, Department of Development and Regeneration, Stem Cell Biology and Embryology Unit, Leuven Stem Cell Institute, Herestraat 49, B-3000 Leuven, Belgium; 10 INSERM UMR1087, CNRS UMR6291, Université de Nantes l’institut du thorax, Nantes, France; 11 INSERM, UMS 016, SFR Francois Bonamy, Cytocell Core Facility, Nantes, France; CNRS, UMS 3556, Nantes, France; Université de Nantes, Nantes, France; CHU Nantes, Nantes, France; 12 CHU Nantes, Service de génétique médicale, Nantes, France; 13 INSERM, UMR1238, Bone Sarcoma and Remodeling of Calcified Tissue, Nantes, France; 14 CRCINA, INSERM, Université de Nantes, Nantes, France; 15 CHU Nantes, l’institut du thorax, Nantes, France; 16 Department of Stem Cell and Regenerative Biology, Harvard University, Cambridge, MA, 02138, USA; Broad Institute, Cambridge, MA 02142, USA.; Harvard Stem Cell Institute, Harvard University, Cambridge, MA 02138 USA; 17 Berkeley Lights Inc., 5858 Horton Street, Emeryville, CA 94608 USA; 18 10x Genomics, 7068 Koll Center Pkwy #401, Pleasanton, CA 94566 USA; 19 Laboratory of Human Genetics of Infectious Diseases, Necker Branch, INSERM U1163, Paris, France; 20 Institut Pasteur, Department of Immunology, Lymphocyte Cell Biology Unit, Paris, France; 21 Department of Clinical Pathology and Cytology, Karolinska University Hospital, Stockholm, Sweden; 22 Institut Pasteur, Dynamics of Immune Responses Unit, 75015 Paris, France; 23 Unit of Antibodies in Therapy and Pathology, Department of Immunology, Institut Pasteur, Paris, France; 24 Unit for Lymphopoiesis, Immunology Department, Pasteur Institute, Paris, France; 25 Laboratory of Dendritic Cell Immunobiology, Department of Immunology, Institut Pasteur, 75015, Paris, France; INSERM U1223, 75015 Paris, France; Center for Translational Research, Institut Pasteur, 75015 Paris, France; 26 Immunobiology of Infection Unit, Institut Pasteur, 75015 Paris, France; 27 Genome Integrity, Immunity and Cancer Unit, Department of Immunology, Paris, France; Department of Genomes and Genetics, Institut Pasteur, 75015 Paris, France; 28 Innate Immunity Unit, Institut Pasteur, INSERM, U1223 Paris, France; 29 Institut Pasteur, Department of Mycology, Molecular Mycology-CNRS URA3012, Paris, France; 30 Unité Microenvironment and Immunity, Institut Pasteur, 75724 Paris, France; 31 Department of Cell Biology and Infection, Institut Pasteur, Paris, France; 32 Global Health Institute, School of Life Sciences, École Polytechnique Fédérale de Lausanne, Lausanne, Switzerland; 33 Unité de Biologie des Populations Lymphocytaires, Department of Immunology Institut Pasteur, and Centre National pour la Recherche Scientifique, URA1961, 75724 Paris, France; 34 Center for Translational Research, Institut Pasteur, Paris, France; 35 Institut Pasteur, Unité Biologie et génétique de la paroi bactérienne, Dept. Microbiologie, Paris, France; 36 Université Paris 13, Sorbonne Paris Cité, Equipe de Recherche en Epidémiologie Nutritionnelle, Centre de Recherche en Epidémiologies et Biostatistiques, Inserm (U1153), Inra (U1125), Cnam, F-93017 Bobigny, France; 37 Laboratoire d’Immunologie clinique, CIC-4218 et Unité INSERM 932, Institut Curie, Paris, France; 38 Institut Pasteur, Unité de Régulation Immunitaire et Vaccinologie, Paris, France; 39 Laboratory of Humoral Response to Pathogens, Department of Immunology, Institut Pasteur, INSERM U1222, 75015 Paris, France; 40 Center of Bioinformatics Biostatistics, and Integrative Biology, Institut Pasteur, 75015 Paris, France; 41 Institut Pasteur, Unit of Cytokine Signaling, Paris, France; 42 Université Paris Descartes et Département d’hépatologie, Groupe Hospitalier Cochin Hôtel-Dieu, Paris, France; 43 Immunoregulation Unit, Institut Pasteur, 75724 Paris, France; 44 Functional Genetics of Infectious Diseases Unit, Department of Genomes and Genetics, Institut Pasteur, 75015 Paris, France; 45 Virus & Immunity Unit, Department of Virology, Institut Pasteur, Paris, France; 46 Institut Pasteur, Imagopole-CITech, 75015 Paris, France; 47 PSL Research University, INSERM U932, Institut Curie, Paris, France; 48 Unité de Génomique Virale et Vaccination, Institut Pasteur, CNRS UMR3569, Paris, France; 49 Department of Immunology, Hôpital Européen Georges Pompidou, Paris, France; 50 INSERM UMR1160, Université Paris Diderot, AP-HP, Hopital St Louis, Paris, France; 51 Center for Translational Research, ICAReB Platform, Center for Translational Research, Institut Pasteur, Paris, France; 52 Laboratory of Human Evolutionary Genetics, Department of Genomes and Genetics, Institut Pasteur, Paris, 75015, France; CNRS URA3012, Paris, 75015 France

## Abstract

Induced pluripotent stem cells (iPSCs) have considerably impacted human developmental biology and regenerative medicine, notably because they circumvent the use of cells of embryonic origin and offer the potential to generate patient-specific pluripotent stem cells. However, conventional reprogramming protocols produce developmentally advanced, or primed, human iPSCs (hiPSCs), restricting their use to post-implantation human development modeling. Hence, there is a need for hiPSCs resembling preimplantation naive epiblast. Here, we develop a method to generate naive hiPSCs directly from somatic cells, using OKMS overexpression and specific culture conditions, further enabling parallel generation of their isogenic primed counterparts. We benchmark naive hiPSCs against human preimplantation epiblast and reveal remarkable concordance in their transcriptome, dependency on mitochondrial respiration and X-chromosome status. Collectively, our results are essential for the understanding of pluripotency regulation throughout preimplantation development and generate new opportunities for disease modeling and regenerative medicine.

## Abstract (web summary)

Derivation of human induced pluripotent stem cells (hiPSCs) produces primed hiPSCs that can in turn be converted to naive hiPSCs. Here, the authors directly reprogram somatic cells to form both naive and primed isogenic hiPSCs and confirm the similarity of naive hiPSCs to their in vivo counterparts.

## Introduction

Pluripotent stem cells (PSCs) possess the unique ability to self-renew and differentiate into all cell types of a fully functional adult, making them invaluable tools to study human development, model diseases and design new regenerative medicine approaches. In mammals, pluripotency exists in at least two states: naive pluripotency that represents the ground state of pluripotency found in the preimplantation epiblast and primed pluripotency that corresponds to cells poised for differentiation found in the post-implantation epiblast^1,2^. To date, the majority of human embryonic stem cell (hESC) lines have been derived and maintained in the primed state, and identifying culture conditions supporting human naive pluripotency has been a major goal for the past decade. Since 2013, several studies have yielded multiple, distinct conditions to induce and maintain naive pluripotency^3–9^. In parallel, significant progresses have been made to characterize the molecular signature of human preimplantation epiblast cells^10–15^, establishing guidelines to assess human naive pluripotency^16^. Collectively, those studies showed that two media supported naive pluripotent stem cells converted from primed cells or derived directly from human embryos, demonstrating hallmarks of human epiblast cells: 5i/L/AF^8,17,18^ and T2iLGö^7,15,19,20^. However, it remains unknown whether naive pluripotency can be induced from somatic cells directly without a primed intermediate, and if so, with sole expression of OKMS (Oct4, Klf4, cMyc and Sox2), like in mouse^21–23^.

Here we present a protocol enabling the parallel derivation of isogenic human induced primed (hiPSCs) and naive (hiNPSCs) pluripotent stem cells. hiNPSCs are reprogrammed using T2iLGö^7,19^ or RSeT. hiNPSCs are benchmarked against the human preimplantation epiblast, the gold standard of human naive pluripotency, at the transcriptomic, metabolic and epigenetic levels. Overall, hiNPSCs derived in T2iLGö medium display remarkable similarities to preimplantation epiblast. Thus, direct somatic cell reprogramming to human naive pluripotency complements the array of assays enabling in-depth analysis of human pluripotency.

## Results

### Reprogramming somatic cells into naive hiPSCs

**Fig. 1:**
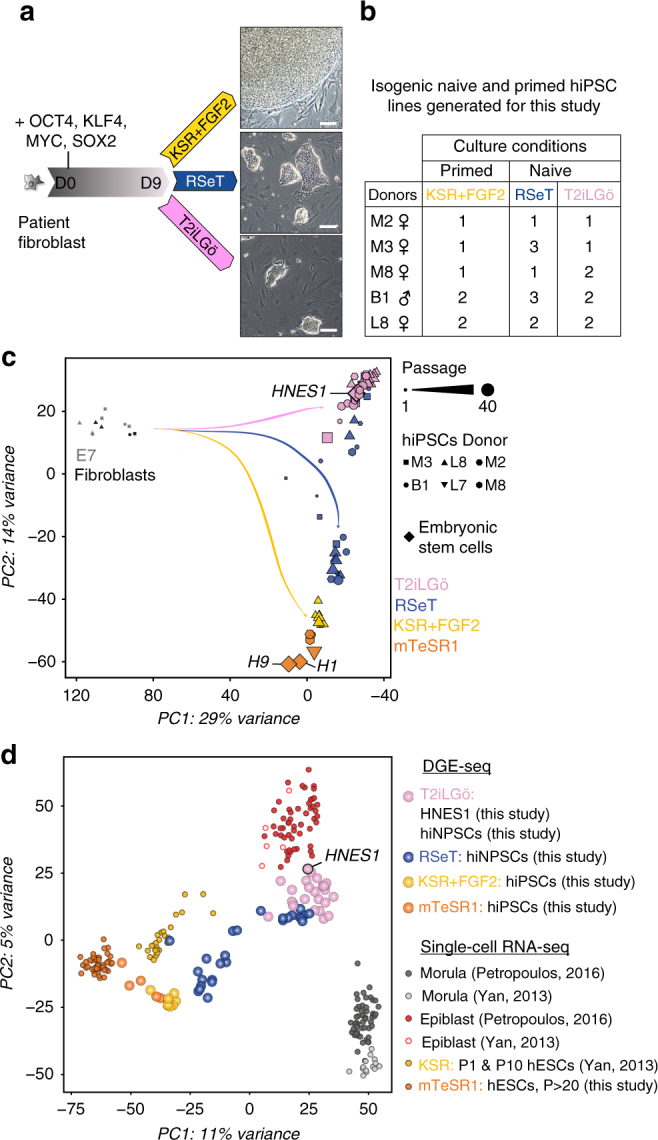
Direct reprogramming of somatic cells into hiNPSCs. **a** Direct generation of isogenic naive and primed hiPSCs. Fibroblasts were transduced with 3 Sendai viruses expressing a polycistron KLF4/OCT4/SOX2, MYC and KLF4 at a ratio of 5:5:3, respectively. Cells were split on feeders at day 7, and placed in the indicated media at day 9. Scale bar = 100 µm. **b** Summary of lines generated for this study in primed (KSR+FGF2, yellow) or naive culture media (RSeT, blue or T2iLGö, pink) originated from 5 different donors. **c** Different pluripotent states are induced depending on culture media. Transcriptomes of hiPSCs and hiNPSCs, control primed hESC lines H1 and H9 or the naive hESC line HNES1^19^ were analyzed by PCA. Symbols represent donor lines, and size of the symbols represents the passage. Arrows have been drawn to highlight the reprogramming trajectories. **d** T2iLGö hiNPSCs are the closest to human epiblast cells. PCA of single-cell RNA-seq data sets from preimplantation embryo samples^11,12^ compared to primed hPSCs from ref. ^11^ and to primed/naive hiPSCs/hESCs from this study

We aimed to develop a direct reprogramming method to simultaneously generate isogenic naive and primed human PSCs. We overexpressed *OCT4, KLF4, MYC* and *SOX2* in human fibroblasts from 5 healthy donors, using a non-integrative Sendai virus. At day 7, cells were split to 3 tissue culture dishes, enabling to induce multiple pluripotent states directly from the same parental cells. At day 9, we cultured emerging colonies in primed pluripotency medium (KSR+FGF2) and in media supporting human naive pluripotency (RSeT and T2iLGö) (Fig. 1a). Both media contain 2i, inhibitors of MEK and GSK3β which are essential for mouse PSCs maintenance^24^, and LIF. Besides 2i and LIF, T2iLGö medium contains a PKC inhibitor^7,19,25^, while the RSeT is a medium derived from the NHSM^5^, composed of inhibitors of JNK and p38, FGF2 and TGFβ1, which supports interspecies chimeras. RSeT medium was chosen due to accessibility and apparent low genomic abnormality rate, and T2iLGö because it was reported to yield cells with more stable genome over 5i/L/AF^7,8,17^. In order to broaden our analysis, we switched some KSR+FGF2 hiPSC lines to mTeSR1 feeder-free medium. In total, we generated 25 cell lines (Fig. 1b and Supplementary Table 1), of which cells grown in RSeT or T2iLGö formed dome-shaped colonies resembling mouse embryonic stem cells (mESCs). We controlled Sendai expression and confirmed transgene independency of hiNPSCs, but at higher passages than in hiPSCs (Supplementary Fig. 1 and Supplementary Table 1). hiPSCs and hiNPSCs display karyotype identical to the parental fibroblasts; however, hiNPSCs tend to acquire chromosomal abnormalities, as previously reported for human naive embryonic stem cells (hNESCs)^8,17,19^ (Supplementary Table 1). These genomic alterations have recently been associated with the inhibition of MEK through PD0325901, one major component of most media supporting human naive pluripotency^26^. We limited the diploid/tetraploid ratio by reprogramming and growing cells under hypoxic conditions and constant rock inhibition (Y27632) (Supplementary Table 1), and by subcloning T2iLGö hiNPSCs.

We analyzed our hiNPSC and hiPSC lines, at different passages, by 3′ digital gene expression RNA-sequencing (DGE-seq), a quantitative method based on molecular indexing of messenger RNA (mRNA) molecules^27,28^. Controls used in this analysis are primed (H1 and H9) and naive (HNES1) hESC lines^19^. Principal component analysis (PCA) revealed two major components: the first discriminating between parental cells and pluripotent stem cells, the second discriminating between H1 and H9 on one side and HNES1 on the other side (Fig. 1c). T2iLGö and RSeT hiNPSC lines were separated along the second principal component. The majority of T2iLGö hiNPSCs clustered with HNES1, regardless of passage number, while cells exposed to RSeT formed two intermediate clusters and tended to become more similar to primed human PSCs (hPSCs) at later passages (trajectory drawn in Fig. 1c). To assess the development stage associated with hiNPSCs in different culture media, we compared them by PCA to the single-cell RNA-seq data sets of hESCs upon derivation (P1 and P10), high-passage hESCs, human epiblast cells and human morula cells^11,12^ (Fig. 1d). The first component classified samples from the morula cells, the cellular fate preceding pluripotency, to high-passage primed hESCs. Strikingly, T2iLGö hiNPSCs cluster together with epiblast cells while RSeT hiNPSCs sit between primed and naive PSCs. Altogether, these data reveal that our protocol can reprogram somatic cells directly to a state resembling the human epiblast, without an intermediate passage in primed media.

### hiNPSCs express markers specific to human epiblast cells

**Fig. 2:**
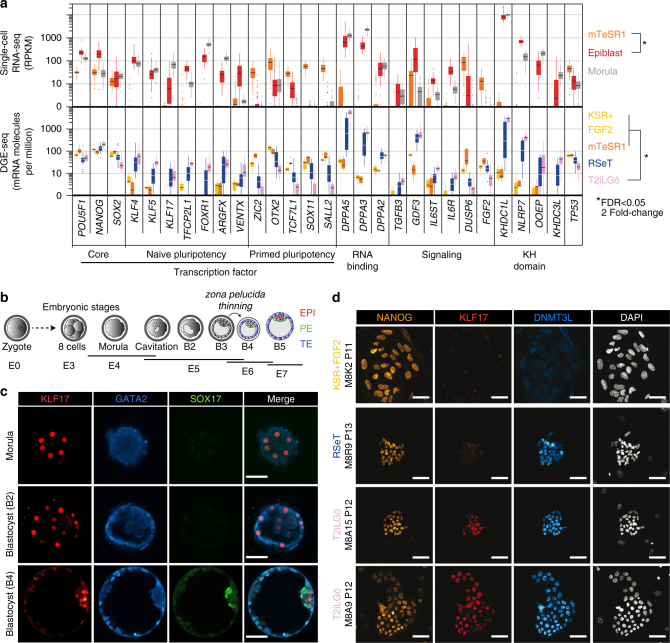
hiNPSCs express markers specific to human epiblast cells. **a** Specific naive pluripotency markers display identical profiles in T2iLGö hiNPSCs and preimplantation epiblast cells. Individual differentially expressed genes plotted as RPKM for single-cell RNA-seq or mRNA molecules per million of total mRNA molecules for DGE-seq. Upper panel: all genes are differentially expressed (Epi vs primed), except *SOX2*; lower panel: all genes are differentially expressed (T2iLGö vs primed), except *POU5F1(OCT4)* and *NANOG*. Error bars are defined as s.e.m. Statistical tests used to compute differentially expressed genes are defined in the “Differential Expression profiling” section of the Methods. **b** Schematic representation of the human preimplantation development comparing clinical staging (Morula, B2, B3, B4 and B5) with corresponding embryonic days (E). EPI epiblast cells in red, PE primitive endoderm cells in green; TE trophectoderm cells in blue. **c** KLF17 protein is expressed in all morula cells before being restricted to epiblast cells in the blastocyst. Human embryos were cultivated in a time-lapse microscope and fixed at indicated stages (morula, B2 or B4 blastocysts). Immunofluorescence for KLF17 (red), GATA2 (blue) and SOX17 (green) was performed. For each indicated embryonic stage, immunofluorescence was performed on 3 biological replicates. Scale bar = 50 µm. **d** KLF17 protein is specifically expressed in T2iLGö hiNPSCs (pink) and not in isogenic lines cultivated in RSeT (blue) and KSR-FGF2 (yellow). Indicated cell lines were analyzed by immunofluorescence for NANOG (yellow), KLF17 (red) and DNMT3L (cyan). This figure is representative of 8 biological replicates. Scale bar = 50 µm

To further characterize hiNPSCs, we compared in depth their transcriptomes (obtained by DGE-seq) to that of human preimplantation epiblast (generated by single-cell RNA-seq)^11,12^. To reduce influence of sequencing protocols, we performed a two-step analysis. We first compared the human epiblast signatures^12^ with those we obtained by performing single-cell RNA-seq from 52 H1 and H9 primed hESCs (Supplementary Fig. 2a). This yielded 6628 differentially expressed (DE) genes when a cutoff of twofold and false discovery rate (FDR) of *<*0.05 was applied. Second, we compared transcriptomes obtained by DGE-seq of primed hESCs and hiPSCs in KSR+FGF2 or mTeSR1 to T2iLGö hiNPSCs, as they clustered with HNES1 (Supplementary Fig. 2a). Using the same cutoff, we found 3003 DE genes between hiPSC and T2iLGö hiNPSC populations, among which 1980 are common DE genes between hESCs and epiblast cells. Among the top DE gene candidates overexpressed in epiblast and hiNPSCs, we found genes related to RNA binding, such as the *DPPA* family or the KH-domain proteins *KHDC1L*, *NLRP7*, *OOEP* and *KHDC3L* (Fig. 2a). Quantitative DGE-seq showed that those genes represent 1% of the transcriptome in T2iLGö hiNPSCs and HNES1, suggesting that mRNA processing regulated by those genes might play a prime role in naive pluripotency. Our analysis further identified transcription factors whose expression was specifically elevated in naive cells, such as *KLF4* and *KLF5*, and a cohort of specific naive pluripotency regulators: *KLF17, FOXR1, VENTX* and *ARGFX*; other transcription factors, such as *OTX2* and *SOX11*, were in contrast elevated in hiPSCs (Fig. 2a). Specific signaling pathways were also linked to pluripotency status. The transforming growth factor-β (TGFB) pathway ligands *TGFB3* and *GDF3* as well as the interleukin-6 (IL6) receptors *IL6R* and *IL6ST* were overexpressed in naive PSCs, while *FGF2* was distinctly overexpressed in primed PSCs.

Among the genes differentially expressed between naive and primed PSCs, we further investigated the extremely high expression of *DPPA5* in T2iLGö hiNPSCs (which represent around 0.2% of total mRNAs). We confirmed the expression of DPPA5 at the protein level by western blot, only in T2iLGö hiNPSCs but not in the RSeT hiNPSCs or hiPSCs (Supplementary Fig. 3a). To our knowledge, it is the first time that DPPA5 protein has been reported as a marker of human naive PSCs. We also investigated the expression of KLF17 at the protein level, in hiPSCs, hiNPSCs and human embryos. During human preimplantation development, pluripotent cells emerge after the morula stage and are restricted to epiblast cells at the blastocyst stage^29^ (Fig. 2b). Immunofluorescence (IF) analysis of human preimplantation embryos revealed that KLF17 is strongly expressed in all cells of the morula (E4/E4.5). KLF17 is also present at the B2 stages (E4.5/E5), and rapidly becomes restricted to the epiblast at the B4 blastocyst stage (E5.5/E6) (Fig. 2c). KLF17 expression is distinct from that of GATA2, a marker of human trophectoderm, expressed after B2 stage, and SOX17, a marker of primitive endoderm, only expressed at the B4 stage. This supports an important role of KLF17 during establishment of pluripotency in vivo, and is in line with single-cell RNA-seq analysis^12^ (Supplementary Fig. 3b). IF analysis of KLF17 and NANOG in primed and naive hiPSCs showed that while all PSCs expressed NANOG, only the T2iLGö hiNPSCs expressed KLF17 (Fig. 2d and Supplementary Fig. 3c). This was further confirmed by flow imaging, highlighting a striking difference in signal intensity and in the number of KLF17-positive cells with nuclear localization between hiPSCs, RSeT and T2iLGö hiNPSCs (Supplementary Fig. 4). In particular, the intensity median of nuclear KLF17 is 2.2 for the T2iLGö hiNPSCs, 1.08 for the RSeT hiNPSCs and 0.79 for the hiPSCs, confirming that the KLF17 profile for RSeT hiNPSCs is closer to hiPSCs (Supplementary Fig. 4B). In contrast, DNMT3L is upregulated in both T2iLGö and RSeT hiNPSCs at the protein level, revealing the intermediate pluripotent state of RSeT hiNPSCs.

In addition to specific individual markers, we identified a strong correlation between naive pluripotency and pathways related to metabolism (Val-Iso-Leu degradation, purine and pyrimidine metabolism), cell cycle (p53 pathway, cell cycle, apoptosis) and cell junctions (adherent and tight junctions, focal adhesion) (Supplementary Fig. 2b). As those pathways are enriched in both epiblast cells and T2iLGö hiNPSCs, it suggests potential cross-talks between metabolic pathways and human naive pluripotency. Nonetheless, characterization of specific active pathways would be complementary to individual markers or to recently proposed transposcriptome profile^30^ to assess the naive nature of human PSCs. We tested the discriminative power of metabolism, cell cycle or cell junction pathways signatures to classify hiNPSCs, and observed that the result was depending on the pathway (Supplementary Fig. 2b). To improve the predictive power of our pathway-based approach, we performed PCA for each pathway and combined the first components to create a three-dimension space, in which we projected our samples. We observed that all the samples were aligned along a common axis, delimited by primed hESCs on one end and naive preimplantation epiblast cells on the other end. Suitably, each sample is clearly classified along this axis, including the RSeT hiNPSCs which have only partially gained expression of components of those pathways and are sitting between primed hiPSCs (in KSR+FGF2 or mTeSR1) and T2iLGö hiNPSCs (Supplementary Fig. 2c).

Altogether, our thorough analysis highlighted specific markers and pathways that characterize naive pluripotency. Moreover, we uncovered a hierarchy of markers distinguishing between T2iLGö hiNPSCs and intermediate RSeT PSCs.

### Metabolic activity of hiNPSCs

**Fig. 3:**
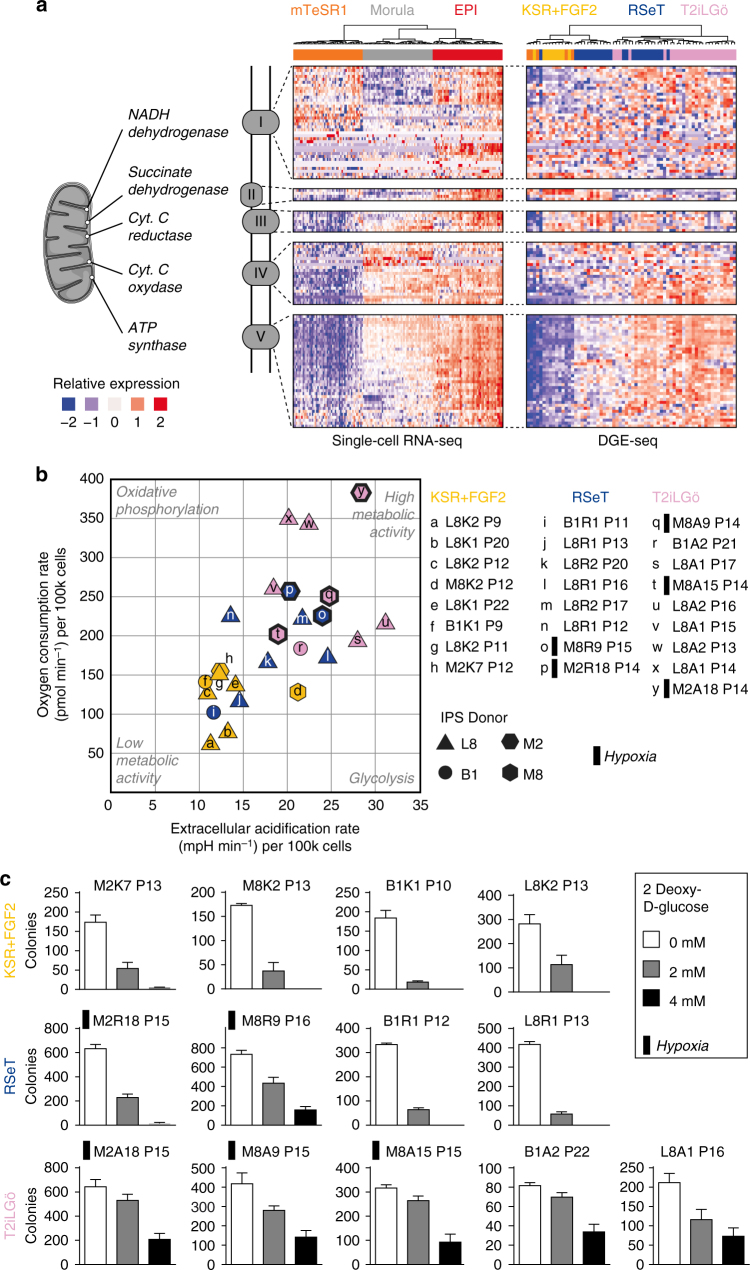
T2iLGö hiNPSCs metabolic profile is closely related to preimplantation epiblast. **a** Genes coding proteins of the electron transport chain, located in the inner membrane of the mitochondria, are upregulated in human epiblast cells and T2iLGö hiNPSCs in comparison to their primed counterparts. Relative expression of genes related to oxidative phosphorylation pathway for hESC, morula and epiblast samples analyzed by single-cell RNA-seq (left), and analyzed by DGE-seq for primed or naive hPSCs (right). Genes were classified by mitochondrion complex and hierarchically clustered. **b** T2iLGö hiNPSCs have higher metabolic activity than their isogenic counterparts in RSeT and KSR+FGF2. A SeaHorse apparatus was used to measure the oxygen consumption rate and the extracellular acidification rate of hiNPSC and hiPSC lines, maintained in indicated culture conditions. This figure presents six biological replicates. Each symbol in the panel is the average of a technical triplicate. **c** T2iLGö hiNPSCs have a higher resistance to inhibition of glycolysis. Quantification of colony numbers obtained after culture with the indicated concentrations of 2-deoxy-D-glucose. Primed cells were seeded in StemMACS™ iPS Brew XF, and naive cells were seeded in the indicated medium. Error bars indicate s.d. of three technical replicates. The presented experiment is representative of four independent experiments

To further characterize naive pluripotency, we analyzed enriched signaling pathways in naive pluripotent cells compared to primed pluripotent cells, based on Gene Ontology (GO) terms and the KEGG (Kyoto Encyclopedia of Genes and Genomes) database^31^, with FDR < 0.01. The top enriched GO terms in hiNPSCs are related to the mitochondrial electron transport chain (Supplementary Data 2) and transcriptomic analysis of genes involved in oxidative phosphorylation shows an overall upregulation in human epiblast cells and hiNPSCs (Fig. 3a). To functionally validate the importance of enriched pathways, we tested the mitochondrial activity by measuring the oxygen consumption rate and the extracellular acidification rates in hiPSCs and hiNPSCs. This showed an increased metabolic activity in naive compared to primed cells, with a combined increase of glycolysis, recently reported in hNESCs^32^, and oxidative phosphorylation (Fig. 3b). Interestingly, the metabolic activity was proportional to the level of naive pluripotency predicted by transcriptomic and pathway analysis: the RSeT hiNPSCs, with a mildly increased expression of electron transport chain genes, had a modest increase in metabolic activity. Moreover, analysis of oxidative phosphorylation capacity showed that hiNPSCs derived in T2iLGö have a higher respiratory capacity than RSeT hiNPSCs or hiPSCs, in line with the analysis of HNES1 cells^19^ (Supplementary Fig. 5). Clonal assays in culture conditions supplemented with 4 mM 2-deoxy-D-glucose, a competitive inhibitor of glycolysis, confirmed the ability of T2iLGö hiNPSCs to mobilize oxidative phosphorylation to proliferate. In contrast, RSeT hiNPSCs and primed hiPSCs did not grow under these culture conditions (Fig. 3c). Therefore, metabolic activity is an important discriminant of hiNPSCs, and could be used to select for naive PSCs.

### Hypomethylation and X-chromosome reactivation in hiNPSCs

**Fig. 4:**
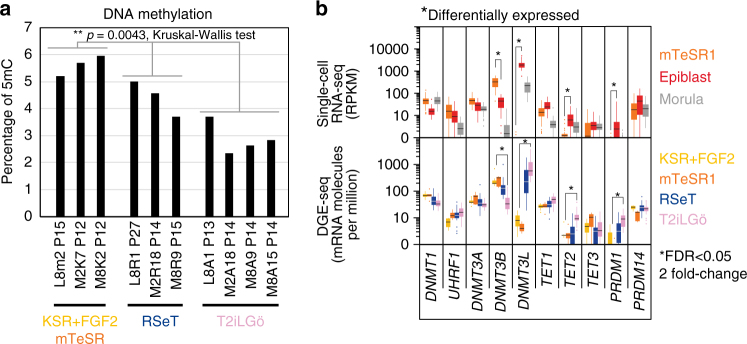
T2iLGö hiNPSCs are hypomethylated. **a** T2iLGö hiNPSCs are hypomethylated in comparison to their RSeT and KSR+FGF2 counterparts. 5mC content is expressed as the percentage of 5mC in the total pool of cytosine for the indicated cell lines. Significance level was determined using Kruskal–Wallis test **p < 0.01. **b** Expression of indicated epigenetic-related genes is plotted as RPKM for single-cell RNA-seq or mRNA molecules per million of total mRNA molecules for DGE-seq. Error bars are defined as s.e.m. *Differentially expressed gene, as defined in Methods

Naive pluripotency is characterized by DNA hypomethylation in human naive PSCs^7,17^ and preimplantation epiblast^13,14^. Quantitation of 5-methyl-cytosine (5mC) by mass spectrometry showed that naive cells in T2iLGö had the lowest mC content of all tested lines, under 3% in average, while primed cells were all above 5%, in accordance with the previously published analysis of hNESC lines^17,19^ (Fig. 4a). Comparison of DNA methylation regulators expression between T2iLGö hiNPSCs and hiPSCs show that *DNMT3L* is dramatically increased (up to 0.1% of the transcriptome) in the former, while *DNMT3B* is decreased. The *TET* family has also been recently associated with human naive pluripotency, as *TET1* overexpression could transiently induce expression of naive markers^33^. Quantitative DGE-seq shows a gain of *TET2* expression in T2iLGö hiNPSCs, whereas *TET1* expression level remains stable and *TET3* is poorly expressed in all samples (Fig. 4b). On another hand, RSeT hiNPSCs had intermediate levels of *DNMT3L, DNMT3B* and *TET2* and 5mC percentage between primed and T2iLGö hiNPSCs. Our results suggest mass spectrometry quantitation of 5mC as a convenient and accurate measurement to qualify hiNPSCs.

**Fig. 5:**
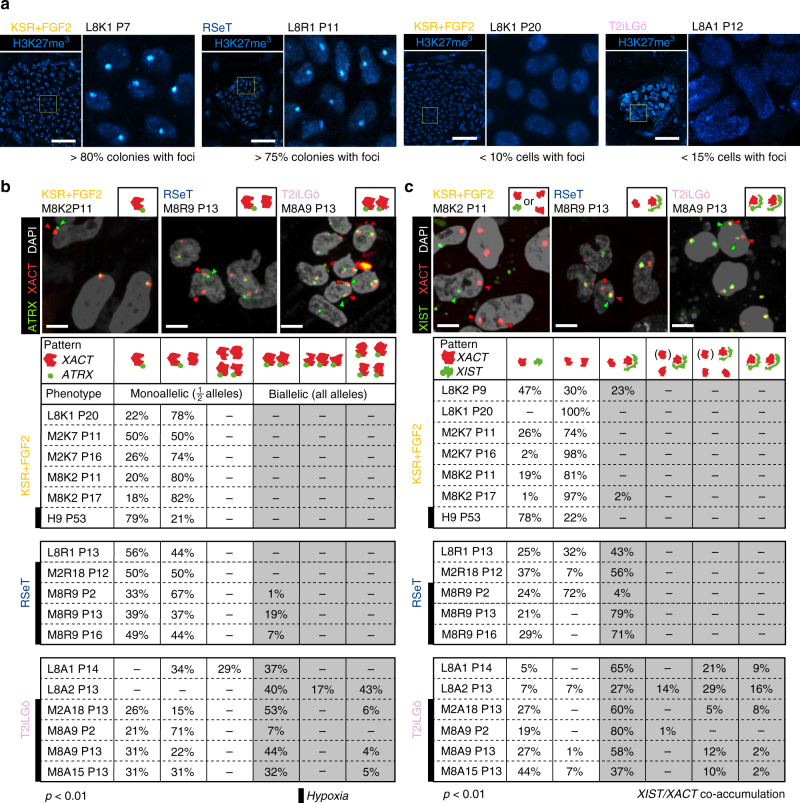
T2iLGö hiNPSCs have a X-chromosome status related to preimplantation epiblast. **a** T2iLGö hiNPSCs or high-passage KSR-FGF2 do not display H3K27me3 foci. Indicated cell lines were analyzed by immunofluorescence for H3K27me3. This experiment is representative of three technical replicates performed at different passages of the cell lines. Scale bar = 50 µm. **b**,** c** T2iLGö hiNPSCs show signs of X-chromosome reactivation. mRNA FISH analysis for **b** *ATRX* and *XACT* or **c*** XIST* and *XACT*. For each cell line represented, more than 100 cells were investigated for their nuclear expression of the indicated mRNA. Quantifications for each combination are indicated below pictures. For each table, samples distribution between FISH counting were found statistically different (*p* < 0.01) by a homogeneity *χ*^2^ test. Scale bar = 20 µm

**Fig. 6:**
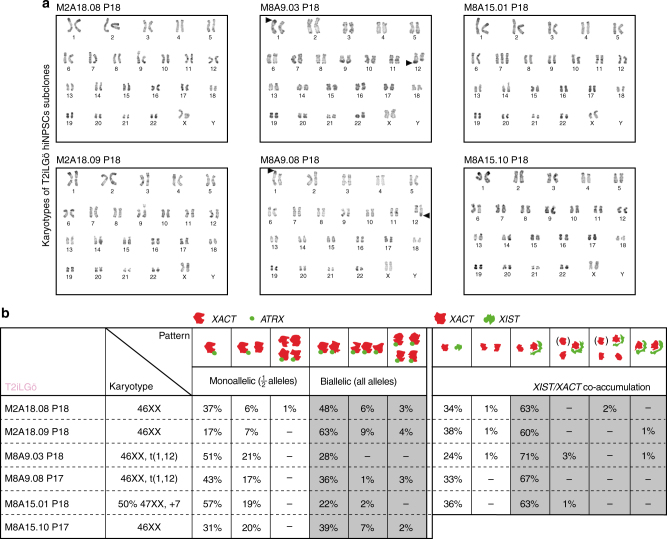
Clonal analysis of X-chromosome reactivation in T2iLGö hiNPSCs. **a** Representative karyotypes of T2iLGö subclones. **b** Karyotype and mRNA FISH analysis for *ATRX* and *XACT* (left) or *XIST* and *XACT* (right) of indicated subclones. For each subclone represented, more than 50 cells were investigated for their nuclear expression of the indicated mRNA. Quantifications for each combination are indicated below pictures

The presence of two active X chromosomes is considered a hallmark of human naive pluripotency^12,18,34^. To assess the activity of the X chromosomes in primed and naive hiPSCs, we first analyzed by IF the distribution of H3K27me3, a marker of the inactive X (Xi) chromosome. Xi-characteristic H3K27me3 accumulation is seen in RSeT hiNPSCs and early-passage hiPSCs nuclei consistent with the presence of an Xi (Fig. 5a). In contrast, a low percentage of T2iLGö hiNPSCs and late-passage hiPSCs display H3K27me3 foci, which could correspond either to the erosion of dosage compensation^35^ that occurs spontaneously in primed hPSCs^36^ or to the reactivation of the Xi, which has been observed in naive PSCs^15,18,30^. To discriminate between the two hypotheses, we monitored by RNA fluorescent in situ hybridization (RNA-FISH) the activity status of the X chromosomes. We first focused on the expression of the protein-coding gene *ATRX*, which has been shown to resist erosion in most studied lines^35,37^. Biallelic expression of *ATRX* is consistently observed in T2iLGö hiNPSC lines, with 36% to 100% naive T2iLGö hiNPSCs displaying only active X (Xa) (Fig. 5b). In contrast, biallelic expression of *ATRX* was rarely observed in RSeT, and never in KSR+FGF2 culture conditions. We next probed the expression of the lncRNAs *XIST* and *XACT*, as their relative patterns of expression clearly distinguish the various X-chromosome states. While early-passage hiPSCs have a characteristic post-inactivation staining, with the Xi coated by *XIST* and the Xa coated by *XACT*, we observed loss of *XIST* and biallelic accumulation of *XACT* in late-passage hiPSCs, further confirming the erosion of X inactivation in these cells (Fig. 5c). In striking contrast, we observed co-accumulation of *XIST* and *XACT* on one active X chromosome in a significant proportion of T2iLGö and RSeT hiNPSCs, but we only observed co-accumulation of *XIST* and *XACT* on both X chromosomes in T2iLGö hiNPSCs, similar to previous findings^15^. Collectively, those results show that X-chromosome reactivation has occurred only in T2iLGö hiNPSCs. To ensure that X-chromosome reactivation was happening independently of chromosomal abnormalities, we subcloned the hiNPSCs M2A18, M8A9 and M8A15. Analysis of two subclones of each line by *ATRX*, *XACT* and *XIST* RNA-FISH demonstrated that X-chromosome reactivation occurs in diploid cells (Fig. 6).

Our FISH results stress out the importance of combining the analysis of *XACT*, *XIST* and *ATRX* expression, and not to rely only on *XIST* or H3K27me3, in order to assess X-chromosome reactivation, a critical hallmark of naive pluripotency in humans^15^ (Fig. 5).

## Discussion

We generated hiNPSCs directly from somatic cells by using OKMS overexpression and defined culture media. Our method enables parallel generation of naive and primed hiPSCs of the same genetic background, limiting tissue culture time and extended passaging compared to previously published strategies that require primed PSCs prior to their conversion into naive PSCs^4–9^. Collectively, our results show that a human preimplantation-like state can be induced in somatic cells by directly shifting reprogramming cultures to naive conditions without the need for a primed intermediate. The resulting hiNPSCs in T2iLGö display all hallmarks of human naive pluripotency, while RSeT hiNPSCs display an intermediate naive phenotype. However, we recorded some genomic alterations in T2iLGö hiNPSCs and others showed aberrant imprinting^17,25^ of the cells. Thus, culture conditions are not yet ideal to maintain in vitro, the transient human naive pluripotent state.

**Fig. 7:**
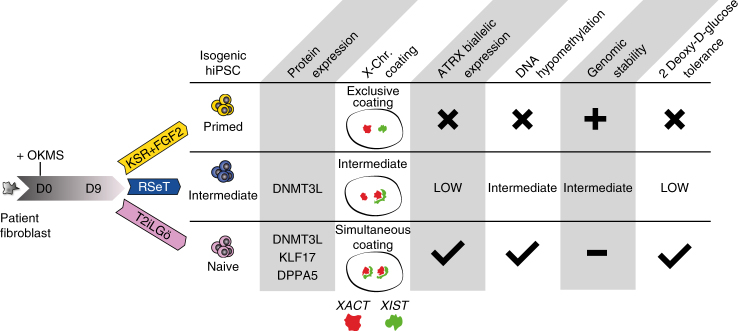
hiNPSCs in T2iLGö achieve the most naive pluripotency hallmarks. The presented reprogramming method enables to simultaneously generate isogenic hiPSCs in KSR+FGF2, RSeT and T2iLGö media. The naive pluripotency level of the generated cell lines can be assessed with specific markers, X-chromosome activity status, DNA methylation level and capacity to tolerate glycolysis inhibition

Further analysis of the array of hiNPSCs that we generated could uncover hierarchy between molecular events necessary to achieve naive pluripotency (Fig. 7). Our data show that transcriptomics analysis is able to rank cells from morula, epiblast/T2iLGö hiNPSCs, RSeT hiNPSCs and primed hESCs/hiPSCs. This supports the concept of a “formative pluripotent state”, a state achieved during the transition from naive demethylated cells to cells primed for differentiation^38^. Our protocol uses OKMS overexpression to achieve a state compatible with naive and primed pluripotency, in line with the first observation of a higher state of human pluripotency^39^. One could envision to use factor-based reprogramming to capture the formative state using proper culture medium.

Besides representing a powerful model to study the differences between naive and primed pluripotency, the multiple metastable states of pluripotency could have significant biological properties. Indeed, one of the approach envisioned for regenerative medicine is to generate interspecies chimeras with human pluripotent stem cells. A recent report showed that specific pluripotent states might be needed depending on the recipient species, limiting the success of the chimeras^40^. During the revision of our manuscript, Yang et al.^41^ described a specific medium allowing generation of pluripotent stem cells with extended potential to contribute to chimeras (EPS cells). However, further characterization is needed to show if those cells correspond to naive pluripotency or have specific features granting them superior chimerism capabilities. In that context, it will be interesting to see how EPS cells qualify using our proposed readouts (Fig. 7).

Altogether, direct reprogramming of somatic cells into hiNPSCs will alleviate ethical issues linked with hESCs, therefore spreading the availability of this important cellular model. Indeed, naive PSCs are considered an alternative to human embryos to study regulation of human pluripotency, model preimplantation development and gonad diseases^42^. A concern for the clinical use of PSCs lies within their ability to keep a stable genome and epigenome, such as the X-chromosome dosage compensation in humans which is deregulated in primed hPSCs after long-term culture and is therefore a potential barrier for regenerative medicine^37^. Combining knowledge obtained from both primed and naive hPSCs will contribute to a better understanding of molecular processes involved in human pluripotency like X-chromosome dynamics, facilitating the development of hPSC-based therapies.

## Methods

### Human preimplantation embryos

The use of human embryos donated to research was allowed by the French embryo research oversight committee: Agence de la Biomédecine, under approval number RE13-010. All human preimplantation embryos used in this study were obtained from and cultured at the Assisted Reproductive Technology unit of the University Hospital of Nantes, France, which are authorized to collect embryos for research under approval number AG11 0126AMP of the Agence de la Biomédecine. Embryos used were initially created in the context of an assisted reproductive cycle with a clear reproductive aim and then voluntarily donated for research once the patients have fulfilled their reproductive needs, or tested positive for the presence of monogenic diseases. All embryos used in this study were given to research after double consents from both parents. Donors did not receive any financial compensation. Molecular analysis of the embryos was performed in compliance with the embryo research oversight committee and The International Society for Stem Cell Research (ISSCR) guidelines^43^.

### Human preimplantation embryos culture

Day 3 cryopreserved embryos were thawed using Sydney IVF Thawing Kit (Cook Medical) and cultured in G2 Plus (Vitrolife), a specific medium for culture of embryos from day 3 to the blastocyst stage. Embryos were loaded into the Embryoscope® (Unisense Fertilitech®), a tri-gas incubator with a built-in microscope allowing time-lapse monitoring of early embryo development.

For embryos affected by a monogenic disease, insemination was achieved by intracytoplasmic sperm injection. Vitrolife® sequential media were used for embryo culture, with embryos being cultured in G1plus® medium from day 0 to day 3 and then transferred to a new pre-equilibrated slide containing G2plus® medium and cultured from day 3 onwards. Embryo biopsy of one or two blastomeres was performed at day 3 and the genetic results were obtained at day 4. Embryo culture was performed at 37 °C under a controlled atmosphere with low oxygen pressure (5% O_2_, 5% CO_2_). Embryos were fixed at the morula, B2 or B4 stages according to the grading system proposed by Gardner and Schoolcraft^44^. Staging details of the embryos that are presented in Fig. 2b were as follows: the morula was fixed at 97 h post fertilization, and contained 24 cells; the B2 blastocyst passed through the morula stage at 59 h post thawing (8 cell stage), and was fixed at 76 h post thawing, it contained 33 cells; the B4 blastocyst passed through the morula stage at 95 h post fertilization, B2 stage at 111 h and was fixed at B4 stage at 118 h, it contained 135 cells, among which 8 PE cells (SOX17+) and 7 EPI cells (KLF17+).

### Human cell lines

Three donor fibroblasts were used in this study, all of them being healthy donors: (1) B1 male fibroblasts are commercial BJ human neonatal fibroblasts extracted from normal human foreskin (Stemgent cat. no. 08-0027), (2) L8 female fibroblasts are normal human adult dermal fibroblasts extracted from a healthy woman aged 57 years, which are commercially available (Lonza cat. no. CC-2511 Lot 0000258580), (3) MIPS female fibroblasts are from three female patients from the Milieu Interieur Labex consortium, M2 and M3 are in their thirties while M8 is in the sixties. Human fibroblasts from the consortium were obtained after informed consent of patients, acknowledging the generation of hiPSC lines and use of those pluripotent lines for research. Primed hiPSCs from L7 human adult dermal fibroblasts extracted from a healthy male aged 51 years (Lonza cat. no. CC-2511 Lot 0000293971) were also used in this study. hESC lines H1 (WA01 Lot WB0111) and H9 (WA09 Lot WB0090) were obtained from the WiCell Research Institute, under authorization RE13-004 from the French embryo research oversight committee, Agence de la Biomédecine.

### Tissue culture

Fibroblasts were cultured in fibroblast medium, composed of high glucose Dulbecco’s modified Eagle's medium (DMEM) GlutamaxII (Life Technologies) supplemented with 10% fetal bovine serum (Hyclone), 1% sodium pyruvate (Life Technologies) and 1% non-essential amino acids (Life Technologies).

Mouse embryonic fibroblasts (MEFs) were prepared as previously described^45^ and cultured in fibroblast medium supplemented with 0.5% of penicillin–streptomycin (Life Technologies). MEF isolation was performed in compliance with the French law and under supervision of the UTE animal core facility, University of Nantes. MEFs were mitotically inactivated using mitomycin C (Sigma) to be used as feeder cells.

Primed PSCs on feeder cells were cultured in DMEM/F-12 (Life Technologies) supplemented with 20% of Knockout^TM^ serum replacement (Life Technologies), 1% non-essential amino acids (Life Technologies), 1% glutamax (Life Technologies), 50 µM 2-mercaptoethanol (Life Technologies) and 10 ng/ml fibroblast growth factor 2 (Peprotech). Primed PSCs were mechanically passaged by cutting colonies with a needle. Primed PSCs in feeder-free conditions were cultured on Matrigel (BD/Corning) in mTeSR1 media; cells were non-enzymatically dissociated with StemMACS passaging solution XF (Miltenyi Biotec) for passaging.

hiNPSCs were cultured on feeder cells, either in RSeT^TM^ medium (Stem Cell Technologies) or in T2iLGö medium^7,19^ which is composed of N2B27 supplemented with 20 ng/ml LIF (Miltenyi Biotec), 1 µM of PD0325901 (Axon Medchem), 1 µM CHIR99021 (Axon Medchem) and 5 µM Gö6983 (TOCRIS). N2B27 medium is composed of DMEM/F-12 (Life Technologies) supplemented with 1% N2 (Life Technologies), 1% B27 (Life Technologies), 1% non-essential amino acids (Life Technologies), 1% glutamax (Life Technologies), 0.1 mM 2-mercaptoethanol (Life Technologies), 50 µg/ml bovine serum albumin (Sigma) and 0.5% penicillin–streptomycin (Life Technologies). hiNPSCs were passaged using TrypLE (Life Technologies) for 5 min at 37 °C.

Naive and primed hPSCs were cultured at 37 °C under 20% O_2_, 5% CO_2_ and 10 µM Y27632 (TOCRIS) was added in the medium upon cell seeding. M2 and M8 naive hPSCs were cultured at 37 °C under 5% O_2_, 5% CO_2_ and 10 µM Y27632.

Somatic cell lines have been tested for mycoplasma presence using the MycoAlert kit (LONZA, LT07-318) before reprogramming. Only if the test was negative, reprogramming was performed. Each iPSC line generated was tested for mycoplasma using the MycoAlert kit at various time points to ensure mycoplasma absence in both primed and naive hiPSCs.

### Reprogramming of human somatic cells into iPSCs

Fibroblasts were reprogrammed using the CytoTune-iPS 2.0 Sendai reprogramming kit from Life Technologies. Two days before infection, 40,000 fibroblasts were seeded per well on a 12-well plate, coated with Matrigel. At day 0, cells were counted and infected with the three vectors: polycistronic Klf4-Oct4-Sox2, cMyc and Klf4 at a 5, 5 and 3 multiplicity of infection, respectively. At day 7 of infection, cells were dissociated using TrypLE and seeded on 3 × 35 mm dishes coated with mouse feeder cells. Cells were switched to naive pluripotency medium (RSeT or T2iLGö) or TeSR-E7 medium at day 9. For each of our reprogramming campaigns, we obtained more than 100 colonies in T2iLGö, TeSR-E7 and RSeT. RSeT and T2iLGö hiNPSCs were trypsinized for passaging, primed hiPSCs were mechanically passaged to fresh feeder-coated tissue culture dishes, in KSR+FGF2, between day 16 and day 24.

### SeaHorse analysis

Oxygen consumption rate and extracellular acidification rate were measured using an XF24 Analyser (SeaHorse Bioscience). At confluence, hiNPSCs and hiPSCs were dissociated using TrypLE and cells were incubated on gelatin for 30 min at 37 °C to remove feeder cells. The SeaHorse plate was pretreated with 2 µg/ml of Cell-Tak Cell and tissue adhesive (Corning). KSR, RSeT^TM^ and T2iLGö cells were seeded at 200,000, 150,000 and 100,000 cells per well respectively prior to the experiment. Cells were incubated for 1 h at 37 °C and atmospheric CO_2_ in DMEM (Sigma Aldrich) supplemented with 10 mM glucose, 2 mM glutamine, 2 mM pyruvate and the pH was adjusted to 7.4 using NaOH. During the mito stress kit experiment, Oligomycin (2 µM), CCCP (0.75 µM), Antimycin-A (2 µM) and Rotenone (1 µM) were injected at indicated time points.

### Colony formation assay in 2-deoxy-D-glucose

hiNPSCs or hiPSCs were seeded at 3000 cells per well in a 12-well plate, coated with feeder cells, in their respective media in addition to 10 µM Y27632. Of note, KSR cells were seeded and cultured in iPS Brew to boost clonogenicity of the cells. From day 1 after seeding onwards, 2 mM or 4 mM of 2-deoxy-D-glucose were supplemented in the culture medium. Cells were fixed between day 4 and day 6 post seeding and stained for alkaline phosphatase using the SIGMA FAST^TM^ BCIP^®^/NBT kit (Sigma). Images were acquired using the Cellomics ArrayscanVTI (Thermo Fisher) at a 5× magnification. Colonies were counted manually. Presented results are from a representative experiment performed in triplicate.

### Karyotype analysis

Karyotyping based on RTG-banding was performed at Cytogenetic Laboratory (CHU Nantes) using standard methods with minor modifications. Briefly, hiPSCs and hiNPSCs were plated on Lab-Tek chamber slide. At 70% confluence, PSCs were submitted to hypotonic shock (20% fetal bovine serum in water), fixed in methanol/glacial acetic acid 3:1, then stained in Giemsa stain. Metaphase spreads for each sample were analyzed. A total of 30 pictures per slides were automatically acquired, chromosomes counted and at least 10 karyotypes for each cell line were classified. The commercial L8 fibroblast line contains a translocation from the chromosome 10 to 7 that is found in all derived iPSC lines. The B1 and M3 fibroblasts had normal karyotypes.

### Immunofluorescence

For IF analysis, cells and embryos were fixed at room temperature using 4% paraformaldehyde for 15 min and 30 min (on a rotating shaker for embryos), respectively. Samples were then permeabilized and blocked in IF buffer (IF buffer: phosphate-buffered saline (PBS)–0.2% Triton, 10% fetal bovine serum) for 60 min at room temperature. Samples were incubated with primary antibodies overnight at 4 °C. Incubation with secondary antibodies was performed for 2 h at room temperature along with 4′,6-diamidino-2-phenylindole (DAPI) counterstaining. Primary and secondary antibodies with dilutions used in this study are listed in Supplementary Table 2.

### Imaging flow cytometry

Cells were stained with the Zombie NIR^TM^ viability kit (BioLegend cat. no. 423105) for 20 min on ice. Potential nonspecific binding sites were blocked by incubation with human serum (obtained from healthy donor at the French blood establishment EFS) diluted to 1:20 in PBS for 30 min. Before performing intracellular staining, cells were fixed and permeabilized for 30 min on ice, using the Fixation/Permeabilization concentrate (eBioscience cat. no. 00-5123) diluted in Fixation/Permeabilization Diluent (eBioscience cat. no. 00-5223) and in the Permeabilization buffer (eBioscience cat. no. 00-8333). Cells were incubated with the primary antibody (anti-KLF17, 1:100) for 45 min at room temperature. Further incubation with the secondary antibody (goat anti-rabbit, 1:500) was performed for 30 min on ice. Before imaging, DAPI was added to stain nuclei of the cells. References of antibodies with dilutions used in this study are listed in Supplementary Table 2.

Analyses were performed using an ImageStreamX Mark II Imaging Flow Cytometer (Amnis Corporation, Seattle, WA) equipped with the INSPIRE software.

A 40× magnification was used for all samples. Data analysis was performed using the IDEAS software (Amnis Corporation). The Zombie NIR® was excited with a 642 nm laser (power 50 mW) and the fluorescence signal was collected on channel 12 (745–800 nm). The DAPI was excited with a 405 nm laser (power 60 mW) and the fluorescence signal was collected on channel 7 (430–505 nm). KLF17 coupled to an Alexa 488 was excited with a 488 nm laser (power 80 mW) and the fluorescence signal was collected on channel 2 (480–560 nm). Intensity-adjusted brightfield images were collected on channel 1 (430–480 nm). The gating strategy for analysis involved the selection of focused, single and living cells on viability marker, then on DAPI and KLF17 fluorescence.

### DNA methylation

For mass spectrometry analysis of DNA methylation, 1 µg of genomic DNA was analyzed using liquid chromatography triple-quadrupole mass spectrometry (KU Leuven Metabolomics Core). The concentration (µM) of Cytosine (unmodified), 5mC and 5-hydroxymethyl-cytosine (5hmC) were obtained using standard curves of known C, 5mC and 5hmC amounts. The percentage of 5mC or 5hmC in DNA was obtained by calculating the ratio of 5mC or 5hmC to the total pool of C.

### RNA-FISH

RNA-FISH was performed as previously described^35^. Briefly, cells were fixed between 36 h and 50 h post seeding in 3% paraformaldehyde for 10 min at room temperature. Cells were permeabilized in CSK buffer supplemented with 1 mM EGTA, 0.5% Triton and RNaseOUT inhibitor (20 U/ml) for 5 min on ice. After 3 washes in 70% EtOH, cells were dehydrated in 90% and 100% EtOH and incubated overnight with probes at 37 °C. After three 50% formaldehyde/2× SSC washes and three 2× SSC washes at 42 °C for 4 min, coverslips were mounted in Vectashield plus DAPI. SpectrumGreen or SpectrumRed-labeled probes (Vysis) were generated by nick translation for human *XIST*, *XACT* (RP11-35D3, BACPAC) and *ATRX* (RP11-42M11, BACPAC Resource). Images were acquired on an inverted Nikon A1 confocal microscope, according to the Shannon–Nyquist sampling rate. mRNA expression of *XIST*, *XACT* and *ATRX* are manually counted in more than 100 cells per cell line.

### Western blot

Cells were lysed in 100 µl of TNTE buffer supplemented with protease inhibitor cocktail (Sigma) and phosphatase inhibitor cocktail (Sigma). TNTE buffer were composed of 50 mM Tris-HCl pH 7.4, 150 mM NaCl, 1 mM EDTA and 0.5% Triton X-100. Then, 20 µg of proteins samples were denatured using NuPAGE sample reducing agent and LDS sample buffer (Invitrogen) for 5 min at 98 °C. Next, 6 µl of spectra^TM^ multicolor broad-range protein ladder (Invitrogen) or 20 µg of denatured protein samples were loaded on a 4–15% mini-PROTEAN^®^ TGX stain-free^TM^ precast gels (BioRad), transferred on trans-blot^®^ turbo^TM^ RTA midi nitrocellulose transfer kit membranes (BioRad). The membranes were blocked for 1 h in tris-buffered saline with Tween-20/5% milk, incubated overnight with primary antibodies and incubated 1 h with secondary antibodies. Signal was revealed with Super signal west femto maximum sensitivity substrate (Thermo scientific) for DPPA5 or clarity^TM^ ECL western blotting substrate (BioRad) for glyceraldehyde 3-phosphate dehydrogenase (GAPDH) and imaged on a Chemidoc^TM^ MP system (BioRad). Primary and secondary antibodies are listed in Supplementary Table 2. The stain-free blot image and the uncropped blot images can be found in Supplementary Fig. 6.

### RNA extraction and quantitative real-time PCR

Total RNA was extracted using RNeasy® columns and DNAse-treated using RNase-free DNase (Qiagen). For quantitative PCR, first-strand complementary DNAs (cDNAs) were generated using 500 ng of RNA, M-MLV reverse transcriptase (Invitrogen), 25 µg/ml polydT and 9.6 µg/ml random primers (Invitrogen).

To quantitate transcripts, absolute quantitative PCR was performed on a Viia 7 (Applied Biosystems) using power SYBR green PCR master mix (Applied Biosystems), for genes listed in the primers table (Supplementary Table 3). For each sample, the ratio of specific mRNA level relative to GAPDH levels was calculated. Experimental results are shown as levels of mRNA relative to the highest value.

All quantitative real-time PCR primers have a hybridization temperature of 60 °C and their sequences are listed in Supplementary Table 3. All amplicons span two adjacent exons. SeV primers are from Life Technologies (cytotune 2.0 kit).

### Expression profiling by single-cell RNA-seq and DGE-seq

For single-cell RNA-seq, H1 and H9 cells were sorted on a FACS Aria in 5 µl lysis buffer (1:500 Phusion buffer, NEB; 1:20 RNASE out, Life Technologies), and frozen at −80 °C. The SmartSeq2 libraries were prepared according to the SmartSeq2 protocol^46,47^ with some modifications^48^. Briefly, total RNA was purified using RNA-SPRI beads. Poly(A)+mRNA was converted to cDNA which was then amplified. cDNA was subject to transposon-based fragmentation that used dual indexing to barcode each fragment of each converted transcript with a combination of barcodes specific to each sample. In the case of single-cell sequencing, each cell was given its own combination of barcodes. Barcoded cDNA fragments were then pooled prior to sequencing. Sequencing was carried out as paired end 2 × 25 bp with an additional 8 cycles for each index.

The FASTQ files were mapped with Tophat2^49^ on GRCH37.75.gtf genome version with Bowtie2^50^ (human_g1k_v37). Of note, FASTQ from^12^ were generated by single-end RNA-seq and in our data set in paired-end RNA-seq. HTSeq^51^ was used to generate raw counts tables from BAM files. For each sample, Q30 percentage was calculated with FASTQC, and samples with a score above 75% were kept. Additional filters were employed: samples with more than 5000 genes detected were kept, and a final gene filtering step was performed to keep genes with a sum of at least 10 counts across the 1976 samples. Samples with total quantity of count 2 s.d. away from mean total quantity of count were excluded.

Counts were normalized with scran^52^ and log2 transformed for variance analysis (PCA and clustering on correlations matrix)

To obtain RPKM, BAM were computed to count using featureCount from Rsubread with GRCH37. Then, counts were normalized with calcNormFactors from edgeR^53^ with default parameters, and RPKM were finally obtained using rpkm function from edgeR on normalized counts. RPKM were used for Figs. 2a and 4b.

For 3′ DGE, RNA-sequencing protocol was performed according to ref. ^28^. Briefly, the libraries were prepared from 10 ng of total RNA. The mRNA poly(A) tails were tagged with universal adapters, well-specific barcodes and unique molecular identifiers (UMIs) during template-switching reverse transcriptase. Barcoded cDNAs from multiple samples were then pooled, amplified and tagmented using a transposon-fragmentation approach which enriches for 3′ends of cDNA. A library of 350–800 bp was run on an Illumina HiSeq 2500 using a Hiseq Rapid SBS Kit v2-50 cycles (ref FC-402-4022) and a Hiseq Rapid PE Cluster Kit v2 (ref PE-402-4002).

Read pairs used for analysis matched the following criteria: all 16 bases of the first read had quality scores of at least 10 and the first 6 bases correspond exactly to a designed well-specific barcode. The second reads were aligned to RefSeq human mRNA sequences (hg19) using bwa version 0.7.4 4 with non-default parameter “-l 24”. Reads mapping to several positions into the genome were filtered out from the analysis. DGE profiles were generated by counting for each sample the number of unique UMIs associated with each RefSeq genes. DGE-sequenced samples were acquired from three sequencing runs. Batch effects were corrected with the limma library function “removeBatchEffect”. All sequenced samples were retained for further analysis.

DESeq2 was used to normalize expression with the DESeq function^54^. Normalized counts were transformed with vst (variance stabilized transformation) function from DESeq library. This log-like transformation was used for variance analysis.

Batch effects were corrected with the limma library function “removeBatchEffect”. All sequenced samples were retained for further analysis. This represents 78 samples cultured from passage 1 to 40 (Supplementary Table 1) in following media: 26 from T2iLGö, 26 RSet, 9 KSR+FGF2, 5 mTESR, 4 fibroblasts and 8 E7.

### Samples assignment of single-cell RNA-seq data

Single-cell samples used in epiblast–hESC comparisons came from two data sets: the first was a subset of 52 hESCs from our own RNA-sequencing, the second was 104 cells from Petropoulos et al.^12^. E4 samples were labeled as morula; epiblast cells were labeled from E5 and E6 blastocysts, after a filtering of cells expressing epiblast markers (Supplementary Table 4). Blastocysts from Yan et al.^11^ were clustered by preimplantation lineage markers (Supplementary Table 4), and a cluster of 5 cells were selected and annotated as epiblast. Two outliers were removed from cells annotated as morula.

For the analysis of KLF17, GATA2 and SOX17 expression profile over development time, samples were stratified per embryonic days. Trophectoderm, and inner cell mass cells were segregated by unsupervised clustering using known lineage markers (Supplementary Table 4). A second clustering was applied on inner cell mass cells to segregate epiblast and primitive endoderm.

### Differential expression profiling

For the DGE-seq data set, differential expressed *p*-values were processed with DESeq2 and FDRs were estimated with Benjamini–Hochberg procedure. For single-cell data set, *p*-values and FDR were computed with ROTS^55^. Genes with FDR < 0.05 and a fold change < 0.5 or > 2 were qualified as differential expressed for both data sets. Two-sided Fisher's exact test was computed to test dependency between single-cell differentially expressed genes and DGE-seq differentially expressed genes with the contingency table found in Supplementary Table 5.

### Processing of principal component analysis

PCAs were computed with R princomp functions from centered data and plotted with R library ggplot2. PCA from Fig. 1d were computed from four data sets: Yan et al.^11^, Petropoulos et al.^12^ and this paper (single-cell and Bulk DGE-seq data). Each data set was transformed into transcripts per million, quantile normalized and *z*-scored by row separately. The data sets were reunified on the 15,315 genes in common for PCA computing.

To generate Supplementary Fig. 2c, a PCA was made for each of the three sets of pathways from Supplementary Fig. 2b (Supplementary Data 2 Tables 3–8). Each single-cell or DGE-seq sample was projected on the first component of the 3 PCA, and the first component coordinates were used to generate the three-dimension graph.

### Processing of heatmaps

Heatmaps were drawn with the library complexHeatmap with *z*-score of expression. Cluster trees were computed with pvclust^56^ with correlation method as distance calculation and Ward criteria as construction method.

For expression profile of KEGG pathways, genes were ordered per fold changes.

### Functional enrichment

topGO^57^ was used to identify enriched GO terms (Supplementary Data 2 Tables 9–10). Enrichment was performed by comparing GO terms present in differentially expressed genes vs. the whole transcriptome data set. Three annotation databases of GO terms were used (org.Hs.eg.db): Molecular Function (MF), Biological Process (BP) and Cellular Component (CC). According to the reference manual, *p*-values were computed with the “classic” and “elim” method algorithm parameter and “Fisher” as statistic parameter.

A gene set analysis method GAGE^58^ was used with KEGG database to identify differentially regulated pathways. Pathways with FDR < 0.01 were retained for further analysis. Gage was used on unpaired mode with parameter “same dir” in false mode.

### Quantification and statistical analysis

All data presented are representative of at least three independent experiments that yielded similar results. Statistical analyses were performed using the software Prism (Graphpad) or R.

DESeq2 was used for analysis considering RNA-seq data were following a negative binomial distribution. Other statistical tests were performed considering their specific assumption and hypothesis, notably for Pearson correlation’s test of Supplementary Fig. 3c and homogeneity *χ*^2^ tests of Fig. 5b, c. All graphical representations were chosen to accurately display variation within each group.

For each experiment, sampling was done to have comfortable group size that provide statistically robust results. For each figure and statistical analysis from RNA-seq data, size of each group is listed in the Supplementary Table 6.

### Data and software availability

The authors declare that all data supporting the findings of this study are available within the article and its supplementary information files or from the corresponding author upon reasonable request.

The raw read sequence data and sample annotations generated for this paper are available at European Nucleotide Archive (ENA) with accession number PRJEB18663.

## Electronic supplementary material

Supplementary Information

Description of Additional Supplementary Files

Supplementary Data 1

Supplementary Data 2
