# Supplementary material for: Parallel derivation of isogenic human primed and naive induced pluripotent stem cells
Source: Nat Commun. 2018 Jan 24;9:360. doi: 10.1038/s41467-017-02107-w (PMC5783949; doi:10.1038/s41467-017-02107-w)
Supplement: Supplementary file 1 — Supplementary Information [file 41467_2017_2107_MOESM1_ESM.pdf]

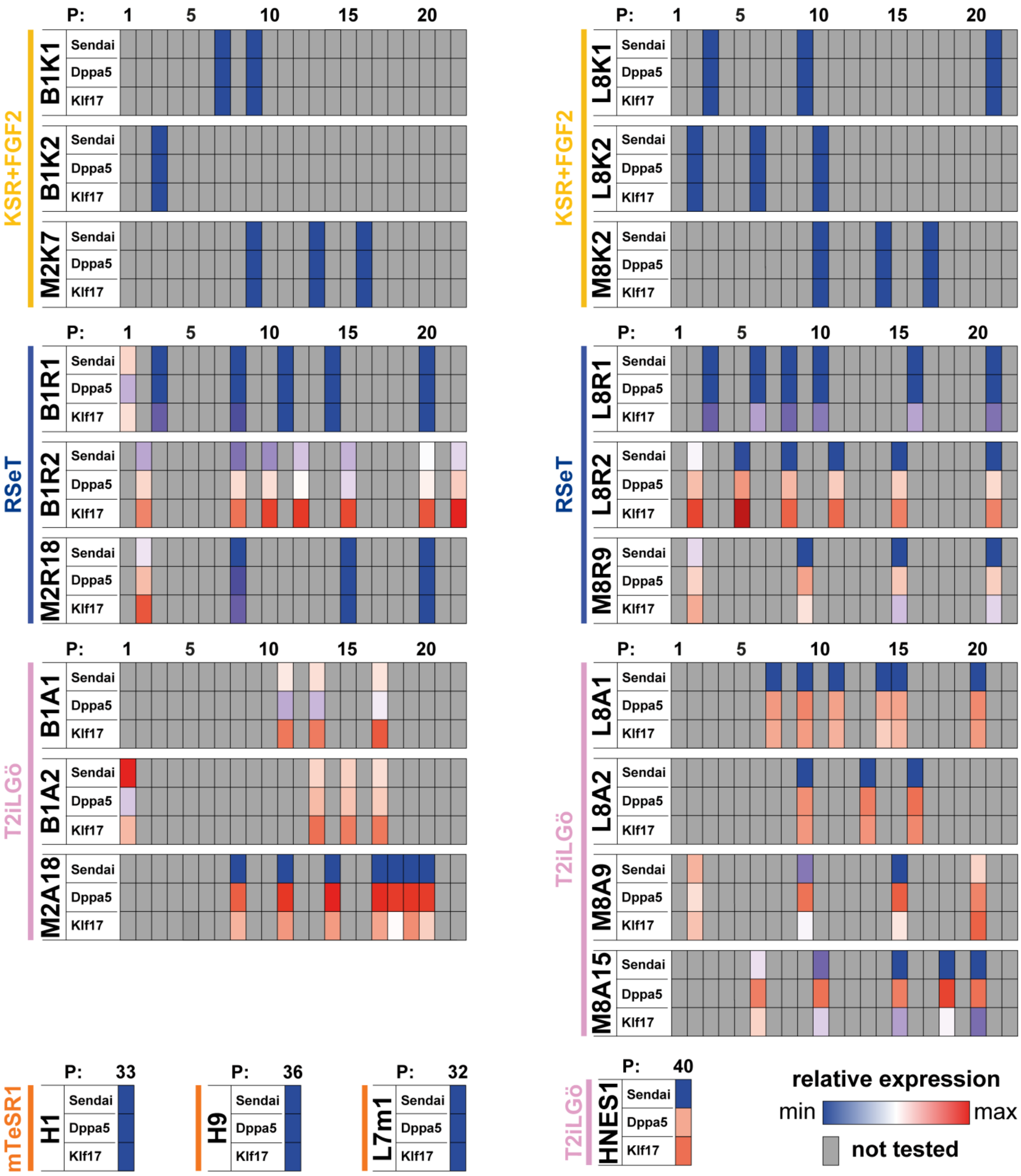

Supplementary Figure 1. **Evaluation of Sendai virus, *DPPA5* and *KLF17* expression across all cell lines** Expression levels of Sendai virus, *DPPA5* and *KLF17* measured by qPCR in indicated cell lines at indicated passages and represented in a blue to red relative scale.

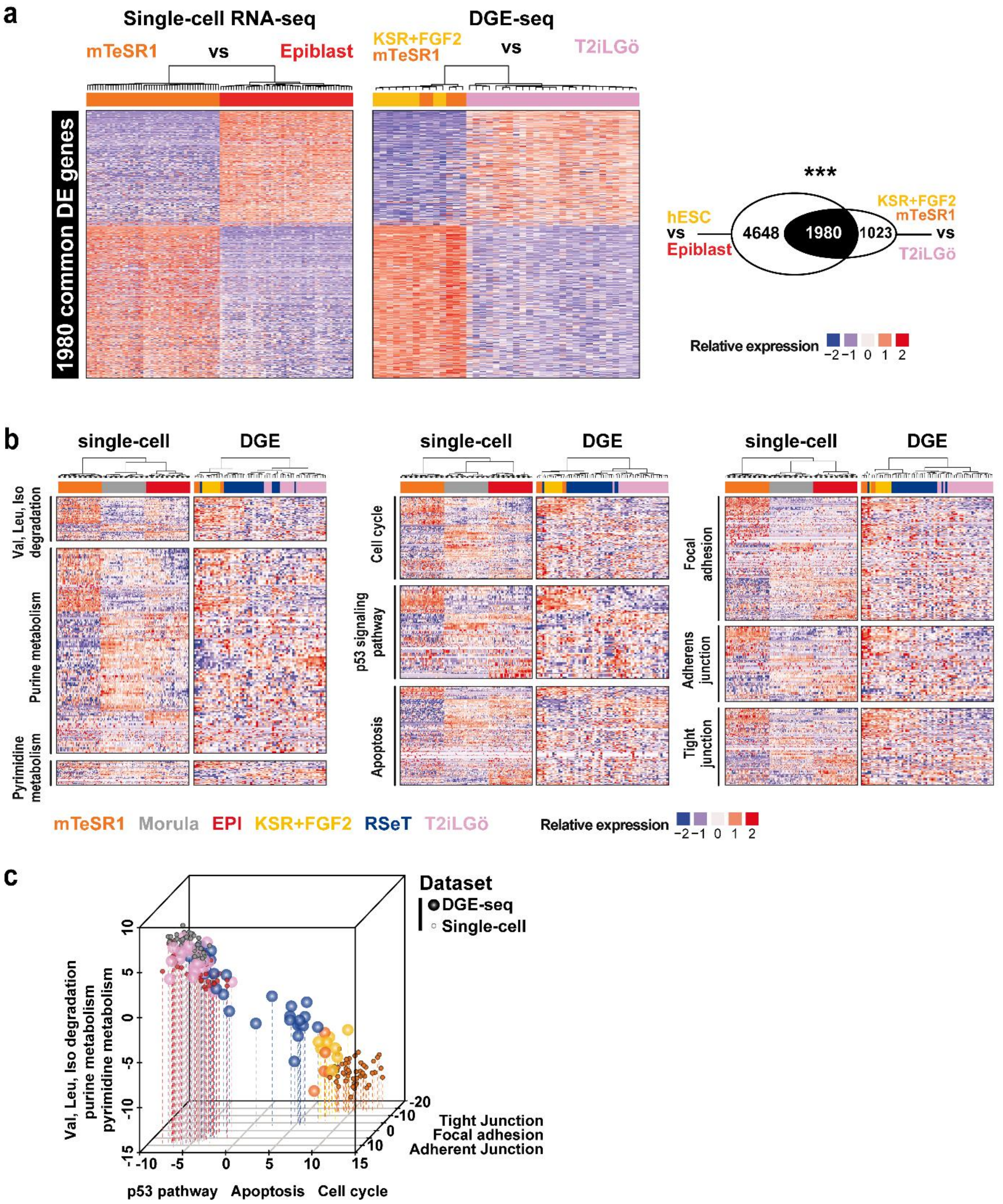

Supplementary Figure 2. **Differential expression analysis and functional enrichment of human epiblast cells and hPSCs**

(**a**) Statistically significant differentially expressed genes, as defined in the Differential Expression profiling section of the Methods, between indicated primed and naive cells, from single-cell RNA-seq on the left and DGE-seq on the right, are represented as a heatmap after hierarchical clustering. Venn diagrams show significant overlaps (Two-sided Fisher exact test, $p<0.01$) in differentially-expressed genes between single-cell and DGE-seq analyses. (**b**) Expression profile of genes associated with significant dysregulated KEGG pathways (GAGE method, FDR<0.01) related to metabolism (Val, Iso and Leu degradation, purine and pyrimidine metabolism), cell cycle (p53 pathway, cell cycle and apoptosis) and cell-cell junctions (adherent and tight junctions, focal adhesion). For each of the three sets of pathways, expression is represented for hESC and epiblast samples analysed by single cell RNA-seq (left), and for primed or naive hPSCs analysed by bulk DGE-seq (right). Genes are classified by pathway then by fold-change. (**c**) Projection of single-cell and DGE-seq samples on the first component of 3 principal component analysis. Each PCA was made on genes from a set of putative dysregulated pathway between primed and naive pluripotency. Each component was found statistically linked with other components by a pearson correlation test ($p<0.01$).

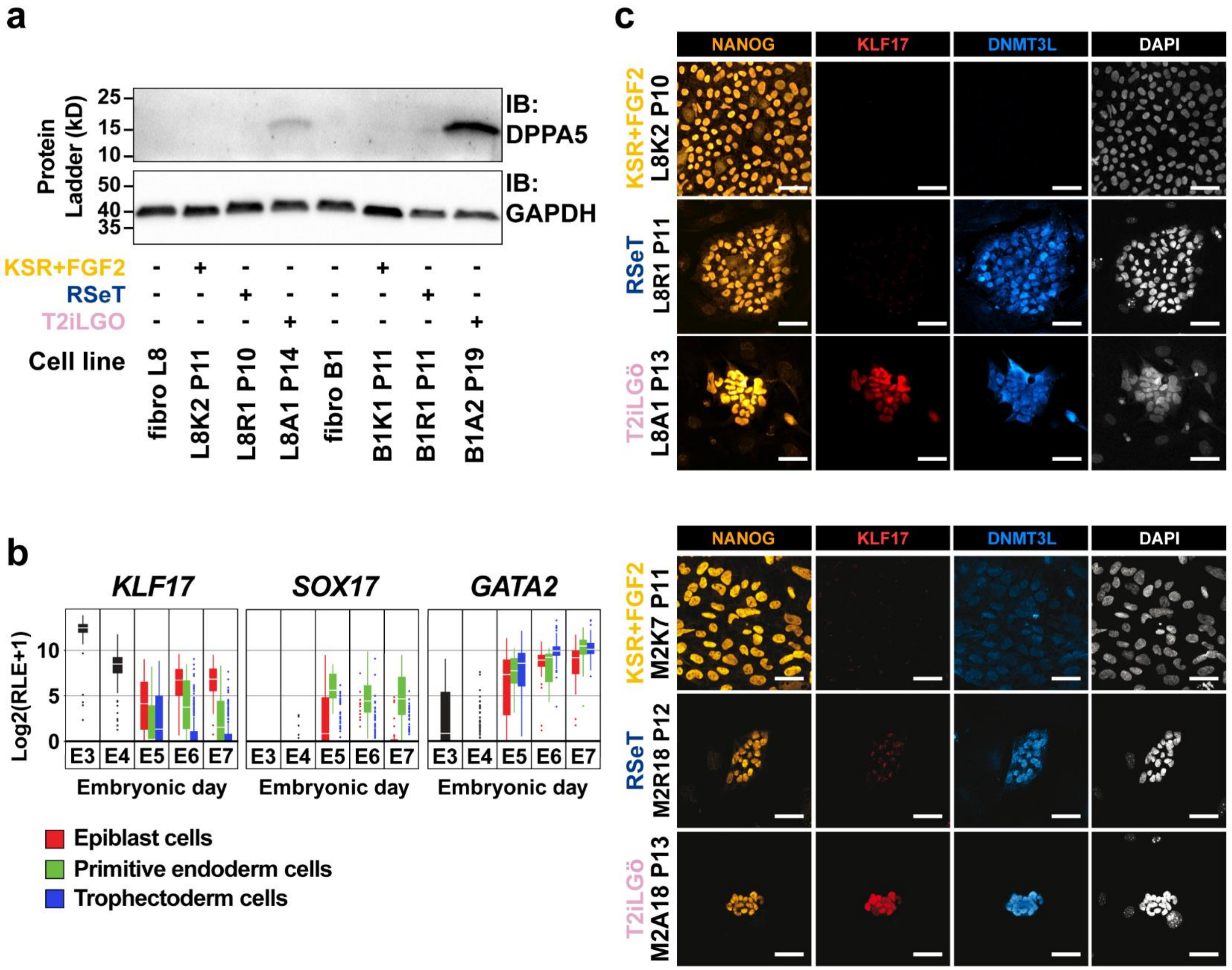

Supplementary Figure 3. **KLF17 and DPPA5 protein expression is restricted to T2iLGö hiNPSCs**
(**a**) DPPA5 protein is only expressed in T2iLGö hiNPSCs in comparison to their RSeT and KSR+FGF2 counterparts. Indicated cell lines were analyzed by western blot for DPPA5 (expected molecular weight: 14kDa) and GAPDH (expected molecular weight: 36kDa). This western blot represents 2 of 3 biological replicates. (**b**) Expression profile of *KLF17*, *SOX17* and *GATA2* in single cells of human blastocysts by embryonic day, and classified by lineage from day 5 onwards using previously known markers (**Supplementary table 4**). RLE = relative log expression. Error bars are defined as s.e.m. (**c**) Indicated hiPSCs were analyzed by immunofluorescence for NANOG (yellow), KLF17 (red) and DNMT3L (cyan). This figure is representative of 5 biological replicates. Scale bar = 50 µm.

a

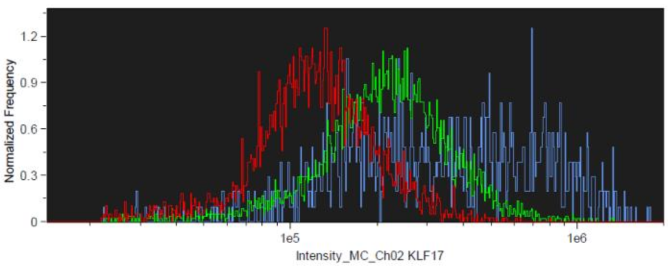

	Cell line	Intensity median
red	M8K2 P16	1.23×10^5
green	M8R9 P15	2.19×10^5
blue	M8A9 P19	3.23×10^5

b

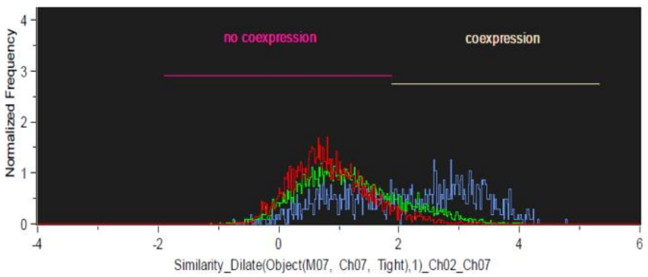

	Cell line	KLF17/DAPI co-localization	Intensity median
red	M8K2 P16	7%	0.79
green	M8R9 P15	19.5%	1.08
blue	M8A9 P19	57.4%	2.20

c

M8K2 P16 - 7% KLF17/DAPI co-localization

M8A9 P19 - 57.4% KLF17/DAPI co-localization

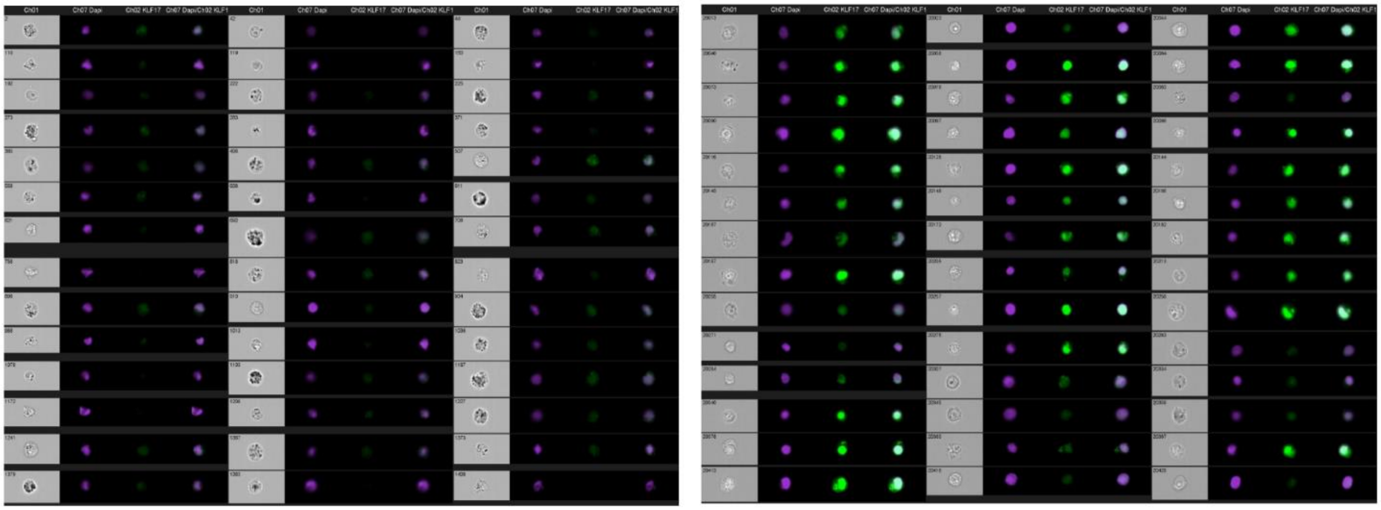

Supplementary Fig. 4. **KLF17 flow imaging confirms intense nuclear expression specifically in T2iLGö hiNPSCs**

Indicated cells were stained for KLF17 and DAPI, and analysed on an image stream X flow imager. Signal was specifically measured from the nuclear region. (**a**) Median value of the signal intensity. (**b**) Percentage of KLF17-DAPI co-localisation and median value of the corrected intensity. (**c**) Image samples from positive hiPSC in KSR+FGF2 (left) or hiNPSC in T2iLGö (right) showing a striking difference in staining intensity.

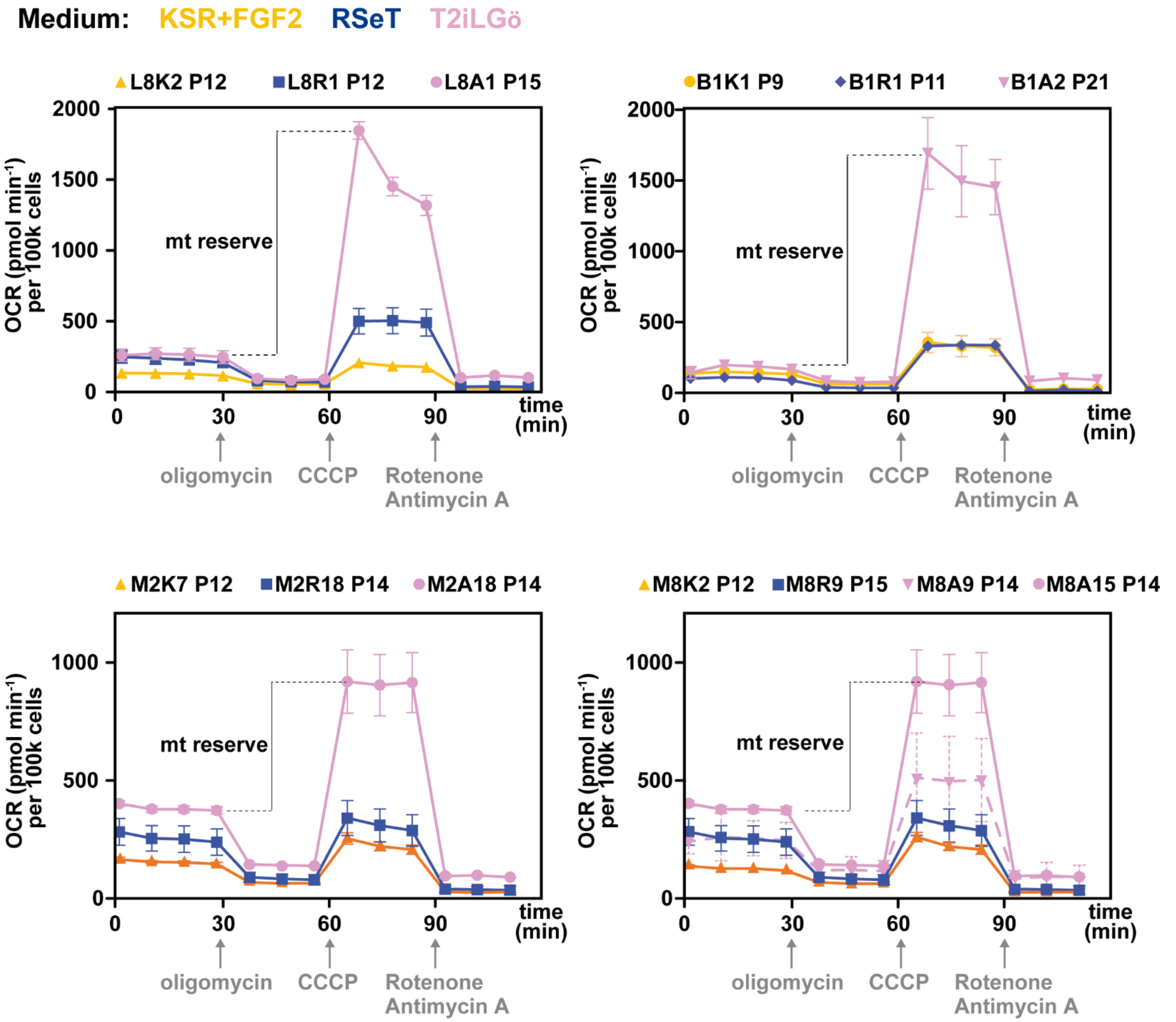

Supplementary Figure 5. **T2iLGö hiNPSCs possess a higher respiratory capacity than RSeT and primed hiPSCs**

Oxygen consumption rate profiles measured by SeaHorse of indicated cell lines. Oligomycin, CCCP and rotenone/antimycin A were injected at indicated time points to evaluate mitochondrial capacity. This figure represents a biological duplicate. Each point of this figure is a technical triplicate and is representative of 5 independent experiments, with s.d. as error bars.

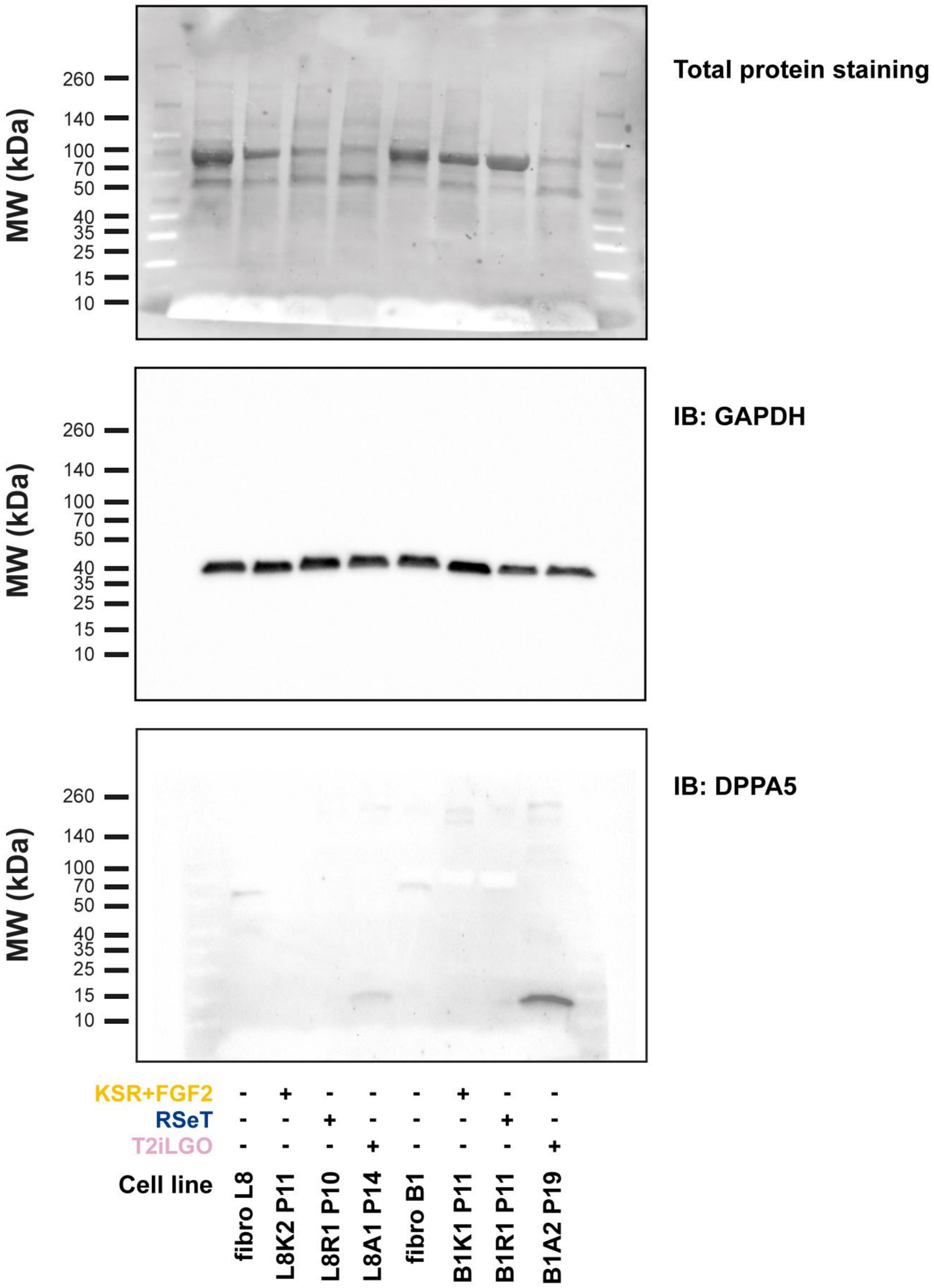

Supplementary Figure 6. **Original Western blot membranes**
From top to bottom images are total protein staining, immunoblot for GAPDH or for DPPA5. For all western blots, Spectra™ Multicolor Broad Range Protein Ladder (Invitrogen) was used.

Supplementary table 1: **Overview of cell lines generated in this study starting from 5 fibroblast cell lines**

	Cell line	Gender	DGE-seq passage in Fig 1B	Sendai	Karyotypic abnormalities acquired	
mTeSR1	H1	♂	P33*	N/A	-	
	H9	♀	P36*	N/A	-	
	L7m1	♂	P32	N/A	none at P30	
	M2m7	♀	P13	neg at P10	none at P12	
	M8m2	♀	P14	neg at P11	none at P12	
KSR+FGF2	L8K1	♀	P3, P7, P9, P16	neg after P3	none at P20	
	L8K2	♀	P6	pos at P2, neg after P6	none at P9	
	B1K1	♂	P9	neg after P7	none at P7	
	B1K2	♂	P3	neg after P3	-	
	M3K1	♀	P4	neg after P3	-	
RSeT	L8R1	♀	P3, P8, P16, P21	neg after P3	none at P10 and P12	
	L8R2	♀	P8, P11	pos at P2, neg after P8	none at P14 and P16	
	B1R1	♂	P1, P3, P8, P20	pos at P1, neg after P3	none at P12	
	B1R2	♂	P2, P8	low until P20, neg at P22	P18: 40% 47,XY,+7 or +5<br>50% other trisomies	
	B1R3	♂	P2, P7	pos at P2, neg after P7	-	
	M3R1	♀	P1, P3, P8	pos at P3, neg after P8	-	
	M3R2	♀	P2, P7	pos at P7	-	
	M3R3	♀	P7	pos at P7	-	
	M2R18	♀	P2, P8	neg after P8	none at P12	**hypoxia**
	M8R9	♀	P2, P9	neg after P9	none at P13	
T2iLGö	L8A1	♀	P7, P9, P11, P15, P20	pos until P9, neg after P11	P14: 50% 92,XXXX	
	L8A2	♀	P9	neg after P9	P13: 12% 92,XXXX; 88% 69,XXX	
	B1A1	♂	-	pos at P17	P16: 100% 92,XXYY	
	B1A2	♂	P11, P13, P17	pos at P21	none at P15	
	M3A1	♀	P20, P25	pos at P25	-	
	M2A18	♀	P8, P11, P14	neg after P8	P14: 67% 92, XXXX	
	M8A9	♀	P2, P9, P15	neg after P15	P13: 25% 46XX, t(1,12)<br>17% 92, XXXX	**hypoxia**
	M8A15	♀	P6, P10, P15	neg after P15	P15: 64% 47, XX, +7;<br>14% 92, XXXX	
	HNES1	♂	P40	N/A	none at P42	

*also in single-cell RNA-seq

N/A stands for not applicable.
A dash means we do not have the data for the particular passage or cell line.

Supplementary table 2: **Antibodies used in this study**

ANTIBODY	SUPPLIER	IDENTIFIER	DILUTION USED
Anti-H3K27me3 mouse monoclonal	Abcam	Cat# ab6002, RRID:AB_305237	1:200
Anti-SOX17 goat polyclonal	R and D Systems	Cat# AF1924, RRID:AB_355060	1:200
Anti-NANOG goat polyclonal	R and D Systems	Cat# AF1997, RRID:AB_355097	1:200
Anti-KLF17 rabbit polyclonal	Sigma-Aldrich	Cat# HPA024629, RRID:AB_1848933	1:500<br>1:100 for ISX
Anti-GATA2 mouse monoclonal	Sigma-Aldrich	Cat# WH0002624M1, RRID:AB_1841726	1:50
Anti-DNMT3L mouse monoclonal	Abcam	Cat# ab93613, RRID:AB_10562109	1:100
Anti-DPPA5 goat polyclonal	R and D Systems	Cat# AF3125, RRID:AB_2094168	1:3000
Anti-GAPDH mouse monoclonal	Santa Cruz Biotechnology	Cat# sc-32233, RRID:AB_627679	1:1000
Zombie NIR™ Fixable Viability Kit	BioLegend	Cat# 423105	1:500
Anti-rabbit Alexa 488	Thermo Fisher Scientific	Cat# A-21206, RRID:AB_2535792	1:1000
Anti-mouse Alexa 568	Thermo Fisher Scientific	Cat# A10037, RRID:AB_2534013	1:1000
Anti-goat Alexa 647	Thermo Fisher Scientific	Cat# A21447, RRID:AB_10584487	1:1000
Anti-rabbit Alexa 488	Thermo Fisher Scientific	Cat# A-11034 RRID:AB_10562715	1:500 for ISX
Anti-goat HRP	Santa Cruz Biotechnology	Cat# sc-2922, RRID:AB_656965	1:5000
Anti-mouse HRP	Santa Cruz Biotechnology	Cat# sc-2055, RRID:AB_631738	1:5000

Antibodies either identified by the catalog number of the supplier or by their Research Resource Identifiers (RRIDs)

ISX stands for Image Stream X

Supplementary table 3: **RT-qPCR Primers used in this study**

Gene Name	Primer sequence 5'-3'	Amplicon size (bp)	Melting Temp. (^oC)	Position
GAPDH	AATCCCATCACCATCTTCCA<br>TGGACTCCACGACGTACTCA	82	80.5	494-576
KLF17	TCAGGAAGGGACTGGTAGAA<br>GTACCCGCATATGTCGTCTAAG	206	83	862-1067
DPPA5	TCCCGAAGACCTGAAAGATCCAGA<br>AATAGGAGCCGTAAACCACGACCT	177	83.5	99-275
SeV	GGATCACTAGGTGATATCGAGC<br>ACCAGACAAGAGTTTAAGAGATATGTATC	181	79	N.A.
NANOG	ATAGCAATGGTGTGACGCAGAAGG<br>CTGGTTGCTCCACATTGGAAGGTT	116	82	701-816

Supplementary Table 4: **Lineage specific makers used to distinguish trophectoderm, epiblast and primitive endoderm cells**

Gene Symbol	Lineage
CDX2	Trophectoderm
CLDN10	Trophectoderm
GATA2	Trophectoderm
GATA3	Trophectoderm
TGFBR3	Trophectoderm
KRT18	Trophectoderm
KRT8	Trophectoderm
EFNA1	Trophectoderm
ARGFX	Epiblast
BMP2	Epiblast
DPPA2	Epiblast
DPPA5	Epiblast
ETV4	Epiblast
FGF4	Epiblast
FOXD3	Epiblast
GDF3	Epiblast
IL6R	Epiblast
KLF17	Epiblast
LEFTY	Epiblast
NANOG	Epiblast
NODAL	Epiblast
OTX2	Epiblast
POU5F1	Epiblast
PRDM14	Epiblast
SOX2	Epiblast
TDGF1	Epiblast
VENTX	Epiblast
ZIC3	Epiblast
GATA6	Primitive endoderm
FOXA2	Primitive endoderm
PDGFRA	Primitive endoderm
BMP6	Primitive endoderm
GATA4	Primitive endoderm
COL4A1	Primitive endoderm

Supplementary table 5: **Contingency table of differentially expressed genes**

Differentially Expressed :	In Single-cell RNA-seq	Not in single-cell RNA-seq
In DGE-seq	1980	1023
Not in DGE-seq	4648	10286

Supplementary table 6: **Sampling size for indicated cell types**

**Dataset**	**Group**	**Sampling size**
Single-Cell	hESC	52
	Epiblast	52
	Morula	52
DGE-seq	T2iLGö (+HNES)	26
	KSR+FGF2	9
	mTeSR1	5
	RSet	26
	E7	8
	Fibroblast	4
