# Supplementary material for: Parallel derivation of isogenic human primed and naive induced pluripotent stem cells
Source: Nat Commun. 2018 Jan 24;9:360. doi: 10.1038/s41467-017-02107-w (PMC5783949; doi:10.1038/s41467-017-02107-w)
Supplement: Supplementary file 2 — Description of Additional Supplementary Files [file 41467_2017_2107_MOESM2_ESM.pdf]

Description of Supplementary Files

File name: Supplementary Data 1
Description: Gene expression in single-cell RNA-seq and bulk DGE-seq datasets. Tab 1-DE-single-cell and Tab 2-DE-DGE represent the expression profile of genes found statistically differentially expressed (DE) in single-cell RNA-Seq dataset (first tab) or DGE-Seq dataset (second tab). Some of those DE genes are presented in Fig. 2a and 4b. Samples are ordered by unsupervised clustering. Genes are ordered by category then by Fold-change. First column corresponds to gene symbol, second indicates if the genes are differentially expressed between single-cell, DGE-Seq or both datasets. Column 3, 4, 5 indicate respectively log2 Fold-Change, adjusted p-value and false discovery rate. Other columns contain the raw expression of each sample. Tab 3-RPKM-single-cell and Tab 4-UPM-DGE represent the quantifiable expression of transcriptomic data and are related to Fig. 2a, 4b. Genes are in rows and samples in columns. Expression is normalised as RPKM for single-cell RNA-Seq dataset (tab 3) and UMI Per Million (UPM) for DGE-Seq dataset (tab 4).

File name: Supplementary Data 2
Description: Gene expression of specific functions in single-cell RNA-seq and bulk DGEseq datasets. Tab 1-Oxphos-single-cell and Tab 2-Oxphos-DGE represent the expression of genes regulating oxidative phosphorylation and are related to Fig. 3a. Genes are in rows and samples in columns. Column 2 indicates which genes belong to each complex. Expression is quantified by log2 normalized counts for single-cell RNA-Seq dataset (Tab 1) and VST RLE for DGE-Seq dataset (Tab 2). Tab 3-Metabolism-single-cell, Tab 4-Metabolism-DGE, Tab 5-CellCycle-single-cell, Tab 6-CellCycleDGE, Tab 7-Junctions-single-cell and Tab 8-Junctions-DGE represent the expression of genes related to dysregulated KEGG pathways between naive and primed pluripotency, related to Supplementary Fig. 2b, 2c. Expression is quantified by log2 normalized counts for single-cell dataset and VST RLE for DGE-Seq dataset. Genes are in rows and samples in columns. Column 2 corresponds to the KEGG pathway associated with each gene. Each tab contains expression of genes linked to one set of related KEGG pathways: Valine, Leucine and Isoleucine degradation, purine metabolism, pyrimidine metabolism for single-cell RNA-Seq dataset (Tab 3) and DGE-Seq dataset (Tab 4), p53 pathway, Apoptosis and Cell cycle for single-cell dataset (Tab 5) and DGE-Seq dataset (Tab 6), Tight Junction, Focal Adhesion and Adherent Junction for single-cell RNA-Seq dataset (Tab 7) and DGE-Seq dataset (Tab 8). Tab 9-TopGO-single-cell and Tab 10-TopGO-DGE represent the enrichment results of the TopGO analysis. Results were computed for the 3 Gene Ontology. The ontology of term is in the column “ont”, with MF for Molecular Function, BP for Biological Process and CC for Cellular Component. Column “GO.ID” corresponds to the Gene Ontology (GO) id of the term, column “Term” to its name. Column “Annoted” gives number of genes related to the GO term in our gene library, column “Significant” gives the number of differentially expressed genes related to the GO term and “Expected” gives the statically expected number of genes if the GO term were not enriched in DE genes. Column “Rank in Fisher” gives the rank for each ontology of the term, for each ontology the top 100 GO terms are represented. Column “Fisher.elim” gives the adjusted p-value for the enrichment test and “Fisher.classic” the raw p-value. TopGO was performed for single-cell RNA-Seq dataset (ninth tab) and DGE-Seq dataset (tenth tab).
